# Combining PARP with ATR inhibition overcomes PARP inhibitor and platinum resistance in ovarian cancer models

**DOI:** 10.1038/s41467-020-17127-2

**Published:** 2020-07-24

**Authors:** Hyoung Kim, Haineng Xu, Erin George, Dorothy Hallberg, Sushil Kumar, Veena Jagannathan, Sergey Medvedev, Yasuto Kinose, Kyle Devins, Priyanka Verma, Kevin Ly, Yifan Wang, Roger A. Greenberg, Lauren Schwartz, Neil Johnson, Robert B. Scharpf, Gordon B. Mills, Rugang Zhang, Victor E. Velculescu, Eric J. Brown, Fiona Simpkins

**Affiliations:** 1 Penn Ovarian Cancer Research Center, Division of Gynecologic Oncology, Department of Obstetrics and Gynecology, University of Pennsylvania, Philadelphia, PA 19104 USA; 2 The Sidney Kimmel Comprehensive Cancer Center, Johns Hopkins University School of Medicine, Baltimore, MD 21287 USA; 3 Department of Cancer Biology, Perelman School of Medicine, University of Pennsylvania, Philadelphia, PA 19104 USA; 4 Department of Pathology and Laboratory Medicine, Perelman School of Medicine, University of Pennsylvania, Philadelphia, PA 19104 USA; 5 Molecular Therapeutics Program, Fox Chase Cancer Center, Philadelphia, PA 19111 USA; 6 Department of Cell, Developmental and Cancer Biology, Oregon Health & Science University School of Medicine, Portland, OR 97239 USA; 7 Gene Expression and Regulation Program, The Wistar Institute, Philadelphia, PA 19104 USA

**Keywords:** Cancer therapeutic resistance, Ovarian cancer

## Abstract

Ovarian cancer (OVCA) inevitably acquires resistance to platinum chemotherapy and PARP inhibitors (PARPi). We show that acquisition of PARPi-resistance is accompanied by increased ATR-CHK1 activity and sensitivity to ATR inhibition (ATRi). However, PARPi-resistant cells are remarkably more sensitive to ATRi when combined with PARPi (PARPi-ATRi). Sensitivity to PARPi-ATRi in diverse PARPi and platinum-resistant models, including *BRCA1/2* reversion and *CCNE1*-amplified models, correlate with synergistic increases in replication fork stalling, double-strand breaks, and apoptosis. Surprisingly, *BRCA* reversion mutations and an ability to form RAD51 foci are frequently not observed in models of acquired PARPi-resistance, suggesting the existence of alternative resistance mechanisms. However, regardless of the mechanisms of resistance, complete and durable therapeutic responses to PARPi-ATRi that significantly increase survival are observed in clinically relevant platinum and acquired PARPi-resistant patient-derived xenografts (PDXs) models. These findings indicate that PARPi-ATRi is a highly promising strategy for OVCAs that acquire resistance to PARPi and platinum.

## Abstract (web summary)

Patients with ovarium cancer frequently develop resistance to platinum chemotherapy and PARP inhibitors (PARPi). Here, the authors show that the combination of PARP and ATR inhibitors increases the therapeutic response in PARPi and platinum resistant ovarium cancer PDX models.

## Introduction

Despite advances in understanding the genetics of high-grade serous ovarian cancer (HGSOC)^1^, standard frontline care remains surgical debulking and platinum-based chemotherapy. With this approach, more than 80% of women with HGSOC recur^2,3^ and over 14,000 die yearly in the United States^4^. In an era focused on targeted strategies exploiting genetic alterations common in HGSOC, revolutionary clinical trials have led to FDA approval of PARP inhibitors (PARPi) in both the frontline setting as maintenance therapy and in the recurrent setting^5–7^. Drug resistance to chemotherapy and PARPi, however, ultimately emerges and many women run out of treatment options and die from disease^2,3^.

HGSOC has unique genetic mutations and copy-number alterations that render it sensitive to synthetically lethal approaches^1,8^. Approximately 50% of HGSOCs exhibit defects in homologous recombination (HR; e.g., *BRCA1*/*2*)^1,8^. Despite initial sensitivity to platinum-based chemotherapy and PARPi, HR dysfunctional tumors eventually acquire drug resistance^9^. Further, while PARPi have moderate activity against *BRCA* mutant (*BRCA*^MUT^) HGSOC, complete responses to PARPi monotherapy are rare (~2–9%) with partial responses being more common (~35%) for recurrent disease^10^. Furthermore, even in responders, patients recur rather rapidly with few durable responses. The other 50% of HGSOCs are HR-proficient^1^ and about 40% of these tumors exhibit increased Cyclin E expression (CCNE1^HIGH^) either by *CCNE1* gene amplification, copy-number gain, or elevated protein expression. These CCNE1^HIGH^ tumors are associated with poor overall survival and platinum resistance^1,11,12^. Overcoming drug resistance is the ultimate obstacle for curing this disease.

Multiple resistance mechanisms to platinum and PARPi have been described. Platinum resistance may emerge due to reduced intracellular drug accumulation, intracellular inactivation of the agent, increased DNA repair, or impaired apoptotic signaling pathways, to name a few^13^. Mechanisms of PARPi resistance can be HR-dependent or independent. HR-dependent mechanisms include restoration of *BRCA* function either through secondary or reversion mutations^14–16^ or restoration of HR by other means (loss of 53BP1, RIF1, REV7, PTIP, Artemis, or the Shieldin complex) that are independent of *BRCA*^17–23^. Mechanisms independent of HR include upregulation of drug efflux pumps^24^, PARP activity alteration^8,25^, loss of PARG^26^, increased stabilization of replication forks^27–30^, RAS pathway activation^31^, and upregulation of the PI3K/AKT pro-survival pathway^32^. Regardless of the mechanism, strategies to overcome resistance and prolong survival are paramount.

Targeting alternative DNA repair pathways using combination strategies is a scientifically rational approach to overcome drug resistance. PARP inhibition impairs the repair of single strand DNA breaks leading to double-strand DNA breaks, which are preferentially repaired by HR^33–35^. PARP is also known to be involved in ligating lagging strand Okazaki fragments within replication forks promoting fork stability^36^. ATR, when activated by replication stress^37–39^, stabilizes replication forks while also activating the S and G2-M checkpoints to allow repair of damaged DNA. In cancers with increased levels of replication stress (defined by slowing or stalling of replication forks, e.g., with *TP53* loss or *CCNE1* amplification), ATR inhibition not only leads to replication fork collapse, but also loss of the G2-M checkpoint, allowing cells with damaged DNA to progress prematurely into M phase, leading to mitotic catastrophe and cell death^37–40^. As such, potent and selective ATR inhibitors (ATRi) such as AZD6738^41^ and M6620^42^ are in phase I/II clinical trials (clinicaltrials.gov). Through targeting the two different fork stabilizing mechanisms, described above, we hypothesize that combining PARP and ATR inhibition (PARPi–ATRi) will lead to increase DNA double-strand breaks and an increase in tumor cell death in cells regardless of HR status.

We previously showed that PARPi treatment activates ATR/CHK1 and ATRi added to PARPi treatment decreased ATR/CHK1 signaling, release of G2/M checkpoint, increased DNA breaks, and tumor regression in *BRCA* deficient in vitro and PDX models^40^. Using our preclinical drug development platform, we established acquired and de novo PARPi and platinum-resistant cell and PDX models encompassing various pathogenic genetic alterations common in the clinic. Here, we report PARPi-resistant cells with varying genetic context and *CCNE1-*amplified models have a significant baseline activation of ATR/CHK1 signaling and sensitivity to ATR inhibition. ATRi moreover, re-sensitizes cells to PARPi resulting in increased replication fork stalling, double-strand breaks, and apoptosis. Herein, we show that targeting ATR in combination with PARPi is synergistic leading to durable and complete responses in a variety of PDX models that harbor different genetic alterations (*BRCA1* reversion mutation and *CCNE1* amplification), all of which exhibit PARPi or platinum resistance. These studies support the use of PARPi–ATRi for the treatment of ovarian cancers that progress on PARPi in the clinic.

## Results

### Genomic instability and increased ATR/CHK1 with PARPi resistance

**Fig. 1:**
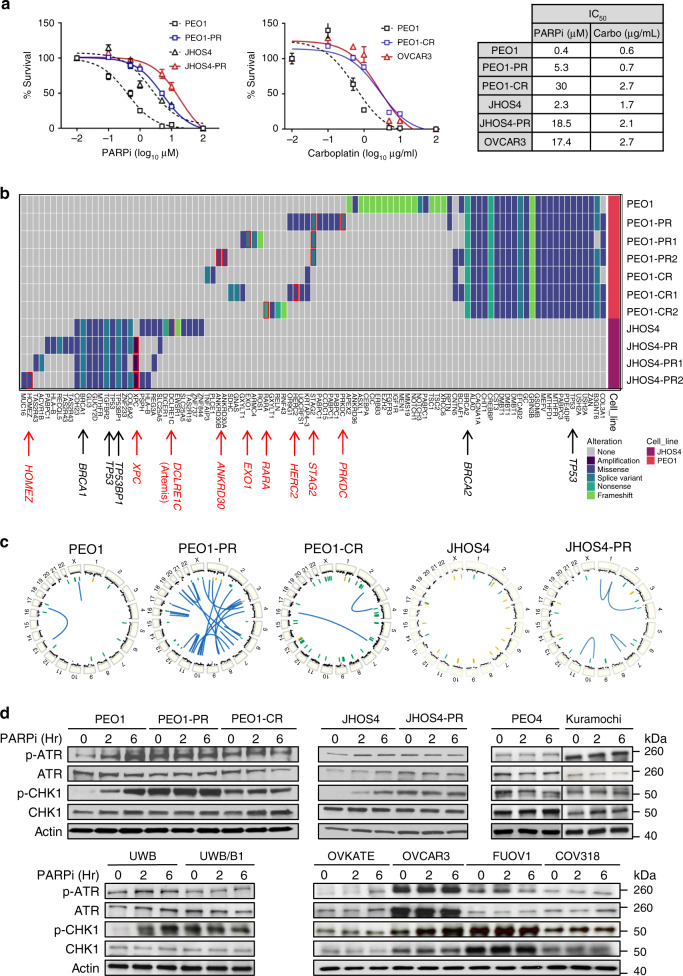
Drug-resistant cells acquire genetic alterations and increase ATR/CHK1 signaling. **a** Drug-response curves of survival after PARPi (olaparib, left) and carboplatin (right) treatment in HR-deficient parental cells (PEO1, *BRCA2*^MUT^; JHOS4, *BRCA1*^MUT^), acquired PARPi-resistant cells (PEO1-PR; JHOS4-PR), and carboplatin-resistant cells (PEO1-CR, and OVCAR3 with *CCNE1*^AMP^) at 5 days. Nonlinear regression curve was generated using MTT data (dose–response inhibition). IC_50_ was calculated by Graph Pad Prism. The fitted midpoints (lC50) of the two curves statistically compared by Extra sum-of squares F test (one-way). Mean ± SD shown (*n* = 3 independent biological replicates per treatment; experiment repeated at least thrice). PEO1 vs PEO1-PR, *P* < 0.0001; JHOS4 vs JHOS4-PR, *P* < 0.0001; PEO1 vs PEO1-CR, *P* < 0.0001; PEO1 vs OVCAR3, *P* < 0.0001. **b** Heatmap of genes and structural alterations present in samples from parental *BRCA*^MUT^ (JHOS4 and PEO1), acquired PARPi-resistant (PEO1-PR, PR1, PR2; JHOS4-PR, PR1, and PR2), and acquired carboplatin-resistant cell line (PEO1-CR, CR1, and CR2). Each column represents a separate alteration by either sequence or structural change, while each row represents a cell line. **c** Circos plots depict copy-number alterations as well as intra- and inter-chromosomal rearrangements. Focal amplifications in yellow, and focal deletions in green. Inter- and intra-chromosomal rearrangements in blue. **d** Parental (*BRCA*^MUT^ PEO1, JHOS4, and UWB; *CCNE1* copy normal, OVKATE), acquired PARPi-resistant (PEO1-PR and JHOS4-PR), de novo PARPi-resistant (PEO4, Kuramochi, and UWB/BRCA1+/−), and platinum-resistant cells (PEO1-CR; *CCNE1*^Amp^ OVCAR3, FUOV1, and COV318*)* were treated with PARPi 1 μM and lysates were collected at 0, 2, 6 h. Cells were selected in PARPi or carboplatin and tested after a 10-day drug washout (except *CCNE1*^AMP^ cells). Western blot for the indicated phospho and total proteins was performed. Representative data shown are one of three independent biological repeat experiments. Source data are provided as a source data file.

PARPi and platinum-resistant (platinum/PARPi-resistant) models were developed from *BRCA1*^MUT^ and *BRCA2*^MUT^ parental cell lines to emulate treatment paradigms used in the clinic, and identify vulnerabilities for therapeutic targets in the context of acquired drug resistance. *BRCA1*^MUT^ (JHOS4-PR, PR1, PR2) and *BRCA2*^MUT^ (PEO1-PR, PR1, PR2) cell lines were developed after long-term continuous treatment (~1.5 years) in olaparib, an FDA approved PARPi. Dose–response curves to PARPi demonstrated a 13-fold increase in the IC_50_ of PARPi-resistant *BRCA2*^MUT^ cells (PEO1-PR) and an 8-fold increase in *BRCA1*^MUT^ cells (JHOS4-PR) when compared with their parental cells (PEO1 and JHOS4, respectively; Fig. 1a). *BRCA2*^MUT^ platinum-resistant cells (PEO1-CR, CR1, and CR2) were developed after long-term treatment with carboplatin (>1 year). PEO1-CR demonstrated a 4.6-fold increase in IC_50_ compared with parental PEO1 (Fig. 1a, Supplementary Fig. 1a).

De novo PARPi resistance was also evaluated. Kuramochi cells (*BRCA2* nonsense mutation (c.6952C>T)^43^ were intrinsically PARPi-resistant despite being *BRCA2*^MUT^ (IC_50_ = 12 μM, Supplementary Fig. 1a). UWB.289/*BRCA1*^+/−^^44^ and UWB.289/*53BP1*^−/−^ were developed from *BRCA1*^MUT^ UWB.289 primary tumor cultures (derived from a g*BRCA1*^MUT^ HGSOC patient) and demonstrated an increase an IC_50_ about 83-fold and 4-fold, respectively, compared with UWB.289 parental (Supplementary Fig. 1a, e).

De novo platinum resistance was studied using PEO4 (*BRCA2*^REV^ mutation)^16^, and platinum-resistant *CCNE1-*amplified (*CCNE1*^AMP^) lines OVCAR3^45^, COV318^46^, and FUOV1^47^, with an increase in IC_50_ of 12.5-fold, 4.5-fold, 23-fold, and 30-fold compared with carboplatin sensitive PEO1 cells (Fig. 1a, Supplementary Fig. 1a). *CCNE1* amplification (*CCNE1*^AMP^) was defined as a copy number >5^48^. Treatment resistance was confirmed by colony formation ability for longer term drug effect assessment (Supplementary Fig. 1b–d).

To characterize the acquired drug-resistant lines and identify new genomic alterations after prolonged drug exposure, PARPi and platinum-resistant cells were evaluated by whole genome sequencing at ~30× coverage (PEO1-PR, PR1, PR2; PEO1-CR, CR1, CR2; JHOS4-PR, PR1, and PR2; Fig. 1b, c, Supplementary Table 1). Interestingly, many altered genes found in the resistant lines were involved in maintaining genomic integrity. Comparing *BRCA2*^MUT^ PARPi-resistant (PEO1-PR) and *BRCA2*^MUT^ platinum-resistant (PEO1-CR; 2 clonal lines for each resistant line), with matched *BRCA2*^MUT^ parental sensitive (PEO1) cells, 16 and 14 new mutations were found, respectively. Genes altered in *BRCA2*^MUT^ PARPi/platinum-resistant lines included *HERC2* (missense change; ubiquitin-dependent DNA repair regulation), *STAG2* (splice variant; sister chromatid separation), *PRKDC* (splice variant; localization of DNA repair proteins), *EXO1* (splice variant; DNA mismatch repair)*, APEX2* (LOH; base excision repair), *EZH2* (LOH; epigenetic regulator of DNA damage checkpoint), and *XRCC6* (LOH; nonhomologous end joining). Alterations in genes related to epigenetic regulation involving transcription factors and regulators (*ANKRD30, RARA)* were also detected. We confirmed there was no functional BRCA2 protein expression in all *BRCA2*^MUT^ PEO1 cell lines (PEO1, PEO1-PR, PR1 and PR2, and PEO1-CR, CR1 and CR2) by western (Supplementary Fig. 1f). All resistant cell lines retained original *BRCA* germline (g*BRCA*) mutations and *BRCA2*^MUT^ cells maintained LOH of *BRCA2* genes (Supplementary Table 1). MDR1 protein efflux pump level was also increased in both PARPi/platinum-resistant *BRCA2*^MUT^ (PEO1-PR and PEO1-CR) cells. MDR1 expression was increased 3-fold and 5.0-fold in PEO1-PR and PEO1-CR cells compared with PEO1. While MDR1 inhibition did not affect PARPi sensitivity in PEO1 parent cells, MDR1 inhibitor partially restored PARPi sensitivity in PEO1-CR cells (both resistant to PARPi and platinum) (Supplementary Fig. 2a, b).

There were eight new mutations found in the acquired PARPi-resistant *BRCA1*^MUT^ (JHOS4-PR) cells compared with isogenic matched *BRCA1*^MUT^ parental cells (JHOS4; Supplementary Table 1). Notable genes altered in *BRCA1*^MUT^ PARPi-resistant lines included *XPC* (amplification; nucleotide excision repair) and *DCLRE1C* (loss of heterozygosity (LOH); nonhomologous end joining). XPC knockdown with siRNA in JHOS4-PR partially restored PARPi sensitivity suggesting functional relevance of this alteration in contributing to PARPi resistance (Supplementary Fig. 2c). Artemis (*DCLRE1C*), a nuclease in the 53BP1 pathway, was mutated in the parent but not in resistant cells. Artemis protein was increased in PARPi-resistant *BRCA1*^MUT^ cells (JHSO4-PR; Fig. 1b, Supplementary Fig. 2a). Because Artemis loss has been reported to cause PARPi resistance, it seems unlikely that reversion of the parental mutation but LOH was a source of PARP-resistance in these cells^21^. Alterations in a gene related to epigenetic regulation (*HOMEZ*) was also detected.

Furthermore, chromosome structural analysis revealed an increase in overall rearrangements and copy-number alterations in resistant lines (PEO1-PR, PEO1-CR, and JHOS4-PR). Intra- and inter-chromosomal rearrangements were specifically increased in acquired PARPi-resistant lines (Fig. 1c). Overall, these data indicated that acquisition of PARPi resistance was accompanied by amplifications and point mutations in a diverse set of genes as well as an increase in chromosome rearrangements.

As PARPi activates the ATR/CHK1 pathway in *BRCA*^MUT^ models^40^, we hypothesized that regardless of potential resistance mechanisms, drug-resistant cells would depend on ATR for survival. Consistent with previous findings, treatment with PARPi increased pATR and pCHK1 in a time-dependent manner in *BRCA1* and *BRCA2*^MUT^ parental cells (PEO1: 4-fold, 5-fold, JHOS4: 2-fold, 5-fold, UWB: 2-fold, 12-fold, respectively, control to 6 h; Fig. 1d). In treatment resistant cell lines, those with acquired resistance to PARPi or platinum (PEO1-PR, PEO1-CR, PEO1-CR1, PEO1-CR2, and JHOS4-PR), and those with de novo PARPi resistance (Kuramochi, UWB/*BRCA1*^+/−^), and platinum resistance (PEO4) exhibited higher basal phosphorylated ATR and CHK1 compared with parental lines (PEO1-PR: 4-, 8-fold; PEO1-CR: 3, 4-fold; JHOS4-PR: 2, 9-fold, UWB/*BRCA1*^+/−^ 1.5, 8-fold, respectively, vs matched parental (Fig. 1d, Supplementary Fig. 2d). Interestingly, increased basal activity was more notable in lines with acquired resistance through PARPi selection than with de novo resistance (e.g., as engineered through BRCA1 ectopic expression), suggesting that mutational changes during the acquisition of resistance might influence increased reliance on the ATR/CHK pathway. Above baseline activation of this pathway was observed in all resistant lines even after a 7–10-day washout after selection, and PARPi after drug washout did not further significantly induce pATR and pCHK1 (Fig. 1d, Supplementary Fig. 2d). Notably, the ATR/CHK1 and DNA repair pathways were two of the most significantly altered in the PARPi-resistant cells (PEO1-PR) confirmed by reverse phase protein array (RPPA; Supplementary Fig. 3a,b).

Basal phosphorylated (pCHK1) and total CHK1 levels were also especially increased in *CCNE1*^AMP^ lines (OVCAR3, FUOV1, and COV318), consistent with high levels of replication stress^49,50^ in these cells compared with *CCNE1* copy normal cells such as OVKATE (pCHK1 increased 4-fold in OVCAR3, 6-fold in FUOV1; and 3-fold in COV318 vs OVKATE, *CCNE1* copy normal cells; Fig. 1d). Given the increase in phosphor-ATR/CHK1 either with acquired PARPi- resistance or from increased CCNE1 expression, these data support targeting the ATR/CHK1 signaling pathway in these models.

### Combination is synergistic in PARPi and platinum-resistant cells

**Fig. 2:**
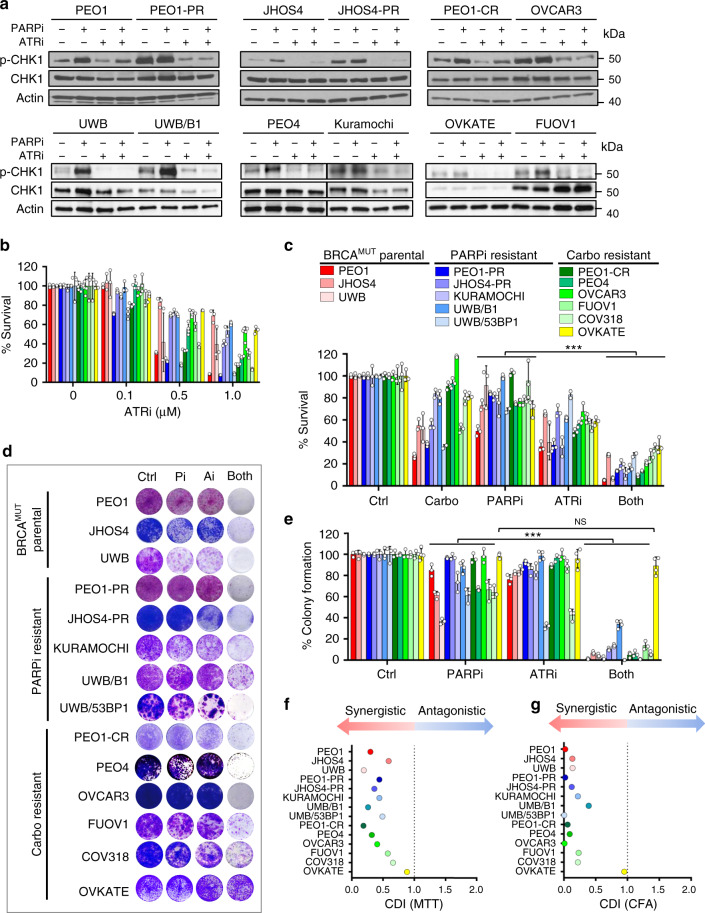
PARPi–ATRi treatment decreases cell viability and colony formation. **a** Western analysis of ATR target, pCHK1/CHK1 in parental *BRCA*^MUT^ (*BRCA2*^MUT^: PEO1; *BRCA1*^MUT^: JHOS4 and UWB), acquired PARPi-resistant (PEO1-PR, JHOS4-PR), de novo PARPi resistant (PEO4, Kuramochi, UWB/B1, B1 denotes *BRCA1*^+/−^), and platinum-resistant (PEO1-CR, PEO4; *CCNE1*^Amp^: OVCAR3, FUOV1 with OVKATE *CCNE1* copy normal) cells after treatment with PARPi (1 μM), ATRi (1 μM), or Both. Representative of three independent biological assays is shown. Band density is normalized to corresponding Actin band (ImageJ). **b** Viability after ATRi treatment in parental *BRCA* mutant, PARPi-resistant cells, and carboplatin-resistant cells by MTT at 5 days. **c** Viability after treatment with carboplatin (1 μg/ml all lines), PARPi (0.1 μM: UWB, UWB/B1, COV318; 0.5 μM: PEO1, PEO4, OVCAR3, FUOV1, JHOS4-PR; 1 μM: PEO1-PR, PEO1-CR, JHOS4, UWB/53BP1, Kuramochi, and OVKATE), ATRi (0.1 μM, UWB/53BP1; 0.5 μM: PEO1, PEO1-CR, JHOS4, COV318, PEO4, PEO1-PR JHOS4-PR, Kuramochi, FUOV1; 1 μM: OVCAR3, UWB, UWB/B1, OVKATE) assessed by MTT at 5 days. Viability in PARPi–ATRi group was lower than PARPi monotherapy for all lines (COV318, OVKATE, *P* = 0.0006, other lines ***P < 0.0001), and ATRi monotherapy for all lines (UWB, Kuramochi, COV318, *P* = 0.04; OVKATE, *P* = 0.008; FUOV1, *P* = 0.0003; remaining lines, *P* < 0.0001). **d**, **e** Colony formation (CF) after treatment with lowest doses demonstrating synergy for PARPi (0.1 μM: PEO1, JHOS4, JHOS4-PR, OVCAR3, UWB, UWB/53BP1; 0.5 μM: PEO1-PR, PEO1-CR, PEO4, FUOV1, Kuramochi, OVKATE; 1 μM, COV318; 2 μM, UWB/B1), ATRi (0.1 μM: JHOS4, UWB, UWB/B1, UWB/53BP1, FUOV1; 0.25 μM, Kuramochi, COV318, OVKATE; 0.3 μM: PEO1-CR; 0.5 μM: PEO4, PEO1, PEO1-PR, JHOS4-PR, OVCAR3) and combination for 13 days; colonies quantified using ImageJ. CF for PARPi–ATRi was lower than PARPi (***P < 0.0001) or ATRi monotherapy for all lines (PARPi–ATRi vs ATRi, UWB 53BP1, *P* = 0.0003; remaining lines, *P* < 0.0001), except OVKATE (PARPi–ATRi vs PARPi *P* = 0.3035, PARPi–ATRi vs ATRi *P* = 0.5073). Data analyzed using one-way ANOVA followed by Tukey’s multiple-comparisons test with data shown as mean ± SD; *n* = 3 biologically independent samples. **f**, **g** Mean % survival and colony formation of a single representative experiment with three determinations was used to calculate coefficient of drug interaction (CDI). CDI < 1 indicated synergism, CDI < 0.7 significant synergism, CDI = 1 additivity, CDI > 1 antagonism. Source data are provided as a source data file.

Given that PARPi and carboplatin-resistant cells have increased baseline pCHK1 levels and that PARPis exert toxicity during S phase, we examined if combining PARPi with ATRi (PARPi–ATRi) would exacerbate basal replication stress responses more so than with ATRi alone. ATRi alone effectively suppressed CHK1 phosphorylation, the downstream effector of ATR kinase, in all cell lines after 24 h (Fig. 2a). Treatment with PARPi increased pCHK1 in all PARPi-sensitive parental *BRCA1/2*^MUT^ cell lines (PEO1: 3.0-fold; JHOS4: 5.0-fold; UWB: 8.4-fold) from baseline more so than in the acquired PARPi/platinum-resistant cells (PEO1-PR: 0.95-fold; JHOS4-PR: 1.4-fold, PEO1-CR: 1.7-fold) and the de novo PARPi-resistant (Kuramochi: 1.2-fold; UWB/*BRCA1*^+/−^: 3.8-fold) and platinum-resistant (PEO4: 2.2-fold, OVCAR3: 1.4-fold, FUOV1: 1.4-fold) where baseline levels were already elevated at baseline at 24 h. Similar to the parental lines (PEO1, JHOS4, UWB), in the acquired and de novo PARPi/platinum-resistant models, the addition of ATRi decreased PARPi-induced CHK1 phosphorylation in all cell lines (Fig. 2a). These data suggest that ATRi added to PARPi would block any potential activation of the ATR/CHK pathway necessary for cell survival.

Given that combination PARPi–ATRi abrogates a critical G2-M checkpoint regulator (pCHK1), cell viability was next assayed. First, using ATRi, AZD6738 at concentrations <1 μM with minimal off-target effects^41^, ATRi monotherapy overall only modestly reduced cell viability in a dose-dependent manner in PARPi/platinum-resistant *BRCA*^MUT^ or restored models, as well as in a *CCNE1*^AMP^ genetic context (Fig. 2b). It appeared, that acquired PARPi-resistant lines (PEO1-PR, PEO1-CR, and JHOS4-PR) were more sensitive to ATRi monotherapy compared with parental, however, the difference was not statistically significant (PEO1, JHOS4; Fig. 2b, Supplementary Fig. 1a, IC_50_ for ATRi). This suggests ATRi alone is insufficient to overcome PARPi/platinum resistance in these models.

We next evaluated whether ATRi treatment re-sensitizes PARPi and platinum-resistant cells to PARP inhibition. Combination PARPi–ATRi was synergistic in decreasing survival and colony formation at drug doses optimized for synergy across all resistant models evaluated (Fig. 2c–g). PARPi–ATRi decreased survival and colony formation ability more so than ATRi alone in acquired and de novo PARPi/platinum-resistant *CCNE1*^AMP^ models (OVKATE *CCNE1* copy normal control; Fig. 2c, d). Overall, both parental and PARPi-resistant *BRCA2*^MUT^ cells were more sensitive to combination PARPi–ATRi than *BRCA1*^MUT^ cells (Fig. 2c, d). Given that drug effects are cell-cycle dependent; longer incubation times may be necessary for optimal drug effects so colony formation ability was evaluated. Combination PARPi–ATRi at low doses (ranges of PARPi 0.01–0.5 μM and ATRi 0.1–0.5 μM) significantly prevented colony formation compared with monotherapy (*P* < 0.05 ATRi vs Both in all cell lines; Fig. 2d, e) and was synergistic with CDI < 0.6 for all cell lines (Fig. 2g). In summary, combination PARPi–ATRi is synergistic in decreasing survival and colony formation in *BRCA1/2* mutant, PARPi/platinum-resistant cell models that acquired resistance to PARPi and platinum or exhibited de novo resistance.

### Cell-cycle drug effects are blunted in drug-resistant cells

Since PARPi–ATRi combination treatment blocks PARPi-induced CHK1 phosphorylation, a key regulator of the G2-M checkpoint, we evaluated their cell-cycle profiles to further understand the mechanism of synergy. As these drugs are cell-cycle dependent, we first assessed the doubling times of all cell lines and observed no significant differences across the lines (mean time 35–44 h; range 35–52 (Supplementary Fig. 4a).

**Fig. 3:**
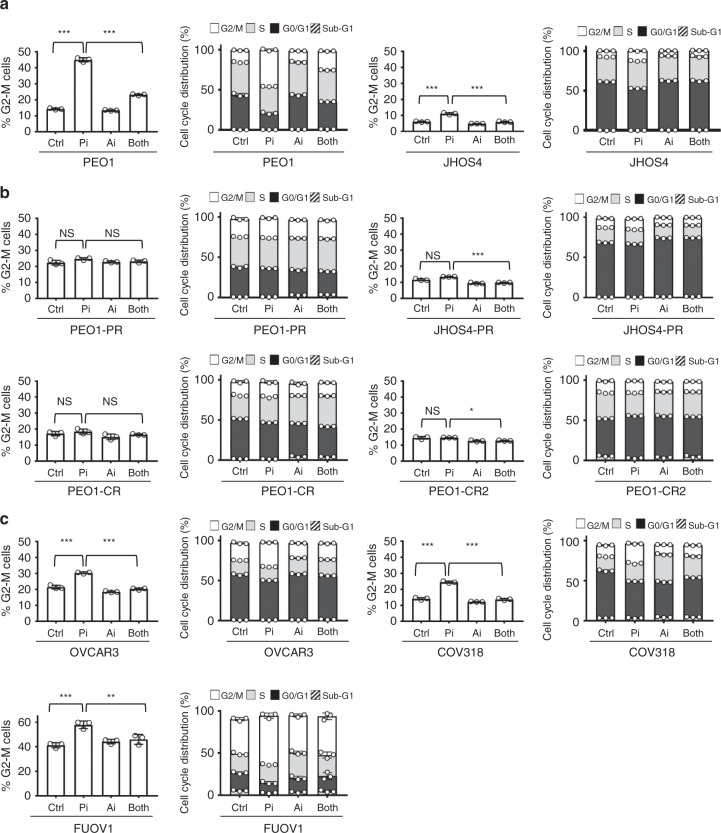
Treatment effects on cell cycle in acquired PARPi and platinum-resistant cells. **a**–**c** Parental *BRCA*^*MUT*^ (PEO1, JHOS4) (**a**), acquired PARPi and platinum-resistant (PEO-PR, JHOS4-PR, PEO1-CR, and PEO1-CR2) (**b**), and platinum-resistant *CCNE1*^AMP^ (OVCAR3, COV318, and FUOV1) (**c**), cells were treated with PARPi (1 μM for PEO1, JHOS4, PEO-PR, JHOS4-PR, PEO1-CR, PEO1-CR2, and OVCAR3; 2 μM for COV318 and FUOV1) and ATRi (1 μM for all cell lines) monotherapy and their combination, and then evaluated for cell-cycle by flow cytometry; G2-M phase changes (left panel) and cell-cycle phase distribution at 24 h (right panel) are shown. In parental *BRCA1/2*^MUT^ PARPi-sensitive cells, there was a significant increase in G2-M with PARPi treatment that was overcome with the addition of ATRi treatment (PEO1 and JHOS4, *P* < 0.0001 Control vs PARPi and PARPi vs Both). In platinum-resistant, *CCNE1*^AMP^ (OVCAR3, COV318, and FUOV1) cells, there was a significant increase in G2-M with PARPi treatment that was overcome with the addition of ATRi (OVCAR3 and COV318, *P* < 0.0001 Control vs PARPi and PARPi vs Both; FUOV1, *P* = 0.0005 Control vs PARPi, *P* = 0.004 PARPi vs Both). In the acquired PARPi and platinum-resistant cells, effects of PARPi treatment on G2/M were insignificant (*P* > 0.05) and with the addition of ATRi, the effects on G2-M were less striking as in parental and *CCNE1*^AMP^ lines (*P* > 0.05 except *P* < 0.0001 for JHOS4-PR and *P* = 0.01 for PEO1-CR2). The data are presented as mean ± SD (*n* = 3 biologically independent samples). Individual samples are presented as data points overlaying bar grafts. The data were analyzed with one-way ANOVA followed by Tukey’s test. ***P < 0.001, **P < 0.01, *P < 0.05, NS = not significant. Source data are provided as a source data file.

In parental *BRCA1/2*^MUT^ PARPi-sensitive cells (PEO1, JHOS4, and UWB), as expected there was a significant increase in G2-M with PARPi treatment by 24 h that was overcome with the addition of ATRi treatment (PEO1 and JHOS4, *P* < 0.001 control vs PARPi and PARPi vs Both; Fig. 3a; Supplementary Fig. 4b). However, in acquired PARPi/platinum-resistant *BRCA1/2*^MUT^ cells there was minimal change in G2-M with either PARPi monotherapy or combination treatment (PEO1-PR, JHOS4-PR, PEO1-CR, and PEO1-CR2 cells, *P* > 0.05 control vs PARPi; Fig. 3b; Supplementary Fig. 4b). In platinum-resistant, *CCNE1*^AMP^ (OVCAR3, COV318, and FUOV1) cells, there was a significant increase in G2-M with PARPi treatment that was overcome with the addition of ATRi (OVCAR3 and COV318, *P* < 0.001; FUOV1, *P* < 0.01; Fig. 3c; Supplementary Fig. 4b). While these data suggest combination drug effects are mediated in part by G2-M checkpoint abrogation in parental *BRCA1/2*^MUT^ and *CCNE1*^AMP^ cells, cell-cycle modulation does not seem to be a significant mechanism driving drug synergy in the acquired PARPi/platinum-resistant, *BRCA1/2*^MUT^ cells.

### ATRi blocks homologous recombination in most resistant cells

**Fig. 4:**
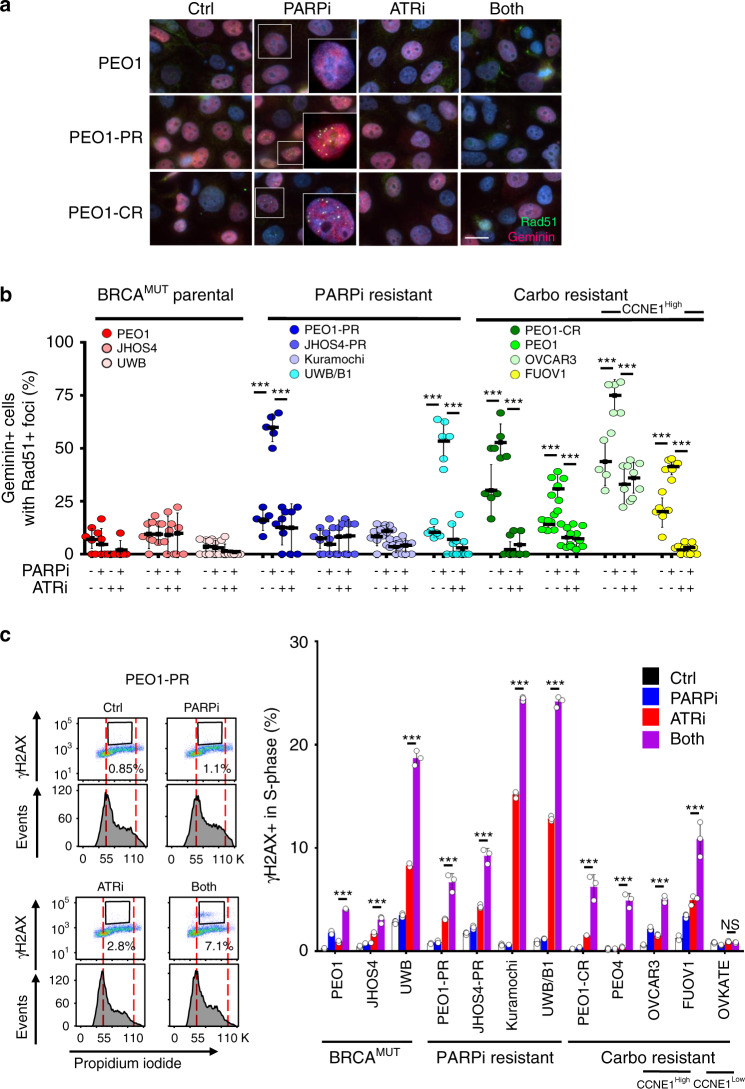
Drug effects on homologous recombination and DNA double-strand breaks. **a**–**c** Parental *BRCA*^MUT^ (*BRCA2*^MUT^ PEO1; *BRCA1*^MUT^ JHOS4, UWB), PARPi-resistant (PEO1-PR, JHOS4-PR, Kuramochi, UWB/B1 where B1 denotes *BRCA1*^+/−^), and platinum-resistant (PEO1-CR, PEO4; *CCNE1*^Amp^ OVCAR3, FUOV1) were treated with PARPi (1 μM), ATRi (1 μM), or combination for 24 h. **a**, **b** RAD51 (green) nuclear foci were detected in geminin positive (red) cells by immunofluorescence staining. Magnification is ×60 for large panel and ×100 for insert. Scale bar = 20 μm. **b** Cells with >5 foci in the nucleus were counted as a positive RAD51 cell. Each dot represents the mean number of events per high-power fields (*n* = 5 fields) with mean ± SD shown. RAD51 in PARPi group was higher than control for PEO1-PR (*P* < 0.0001), UWB/B1 (*P* < 0.0001), PEO4 (*P* < 0.0001), FUOV1 (*P* < 0.0001), PEO1-CR (*P* = 0.002), and OVCAR3 (*P* = 0.0003) lines, and RAD51 in PARPi–ATRi treatment group was lower than PARPi monotherapy for these cell lines (*P* < 0.0001). **c** Detection of ${\upgamma}$H2AX-positive cells in S phase of PARPi–ATRi-treated parental *BRCA*^MUT^, PARPi-resistant, and platinum-resistant cells. Cells were treated with 1 μM PARPi, 0.5 μM ATRi or combination, and JHOS4-PR and OVCAR3 were treated with 1 μM PARPi, 1 μM ATRi, or combination. After treatment for 24 h (JHOS4-PR 36 h), cells were fixed and stained with ${\upgamma}$H2AX and PI for flow cytometry, and ${\upgamma}$H2AX-positive cells in S phase were quantified. Representative images of PEO1-PR cells (left) and quantified data (right) were shown. ${\upgamma}$H2AX-positive cells in PARPi–ATRi group was higher than ATRi monotherapy for all cell lines (JHOS4, *P* = 0.0003; FUOV1, *P* = 0.0002; and remaining cells, *P* < 0.0001) except OVKATE, *P* = 0.3703). Data shown is mean ± SD (*n* = 3 biologically independent samples). Individual samples are presented as data points. Data was analyzed by one-way ANOVA test followed by Tukey’s multiple-comparisons test; ***P < 0.001, NS = not significant. Source data are provided as a source data file.

To further understand the mechanism of drug synergy, effects on HR and accumulation of DNA double-strand breaks (DSB) were next studied. As restoration of HR is one of the major mechanisms of PARPi resistance^14–16,51–54^, we tested this possibility in treatment resistant cells. Rad51 nuclear foci are a biomarker of functional HR and PARPi resistance in *BRCA*^MUT^ breast cancers^52,55^. Here, Rad51 (positive cell was defined as >5 foci/cell) was used as a marker for HR while geminin was used to identify cells in S and G2 phases of the cell-cycle where HR is most prevalent. As expected, there were no Rad51 foci with PARPi treatment in HR-deficient, *BRCA1/2*^MUT^ (PEO1, JHOS4, and UWB) cells (Fig. 4a, b). However, in acquired and de novo PARPi/platinum-resistant cells, RAD51 foci formation in geminin positive cells significantly increased with PARPi treatment indicating HR restoration (PEO1-PR, UWB/*BRCA1*^+/−^, PEO1-CR, PEO4, *P* < 0.001 for Ctrl vs PARPi; Fig. 4b). Interestingly, PARPi-resistant *BRCA1*^MUT^ JHOS4-PR, and Kuramochi cells did not form RAD51 foci upon PARPi treatment, suggesting that the mechanism of PARPi resistance in these lines is independent of HR reconstitution. Notably, RAD51 foci was significantly increased by PARPi treatment in *CCNE1*^Amp^ HR-proficient platinum-resistant cells (OVCAR3, FUOV1, *P* < 0.001 Ctrl vs PARPi; Fig. 4b).

Since ATR contributes to HR in part by promoting Rad51 loading onto DSB^56,57^, we tested the ability of ATRi to block PARPi-induced Rad51 foci in PARPi/platinum-resistant cells. Indeed, ATRi treatment significantly reduced the PARPi-induced RAD51 foci formation in geminin positive cells in PARPi-resistant (PEO1-PR, UWB/*BRCA1*^+/−^) and platinum-resistant cells (UWB/*BRCA1*^+/−^, PEO1-CR, PEO4, OVCAR3, and FUOV1, *P* < 0.001; Fig. 4a, b). While these results do not explain the mechanism of ATRi-mediated cell killing in PARPi and platinum-resistant cell lines that do not regain HR function (JHOS4-PR and Kuramochi), they are consistent with the model whereby ATRi sensitizes PARPi/platinum-resistant cells that have acquired HR capacity by impairing HR^57^.

Since HR restoration would allow DNA DSB repair, we also examined the presence of DSBs by monitoring DSB marker, ${\upgamma}$H2AX in S phase via flow cytometry after 24 h of drug treatment, a time in which we do not yet see apoptosis (Fig. 4c, Supplementary Fig. 5). Indeed, PARPi/platinum-resistant cells accumulated less γH2AX in S phase with PARPi when compared with its parental HR-deficient counterpart (γH2AX-positive cells changes in PAPRi/platinum-resistant cells *P* > 0.05). ATRi monotherapy increased the γH2AX-positive cells more so in all PARPi/platinum-resistant cells compared with matched parental sensitive cells (γH2AX increases in PEO1-PR, JHOS4-PR, and UWB/*BRCA1*^+/−^, *P* < 0.001; PEO1-CR, *P* = 0.098; PEO1, JHOS4, and UWB, *P* < 0.001; OVKATE, *P* > 0.05). However, when ATRi was added to PARPi, γH2AX-positive cells in S phase were significantly increased compared with ATRi alone in all acquired and de novo PARPi/platinum-resistant *CCNE1*^AMP^ models. PARPi–ATRi induced more γH2AX-positive cells in PARPi-resistant cells than matched parental cells (PEO1-PR, JHOS4-PR, UWB/*BRCA1*^+/−^, Kuramochi, PEO1-CR, PEO4, OVCAR3, FUOV1, PEO1, JHOS4, UWB, all lines with *P* < 0.001; except OVKATE, *P* > 0.05; Fig. 4c). Collectively, the mechanism of PARPi–ATRi synergy may include suppression of RAD51 recruitment when maintained or reacquired, but other mechanisms may be possible when reacquisition of RAD51 accumulation does not accompany PARPi resistance (e.g., JHOS4-PR and Kuramochi).

### PARPi–ATRi synergistically decreases fork speed and asymmetry

**Fig. 5:**
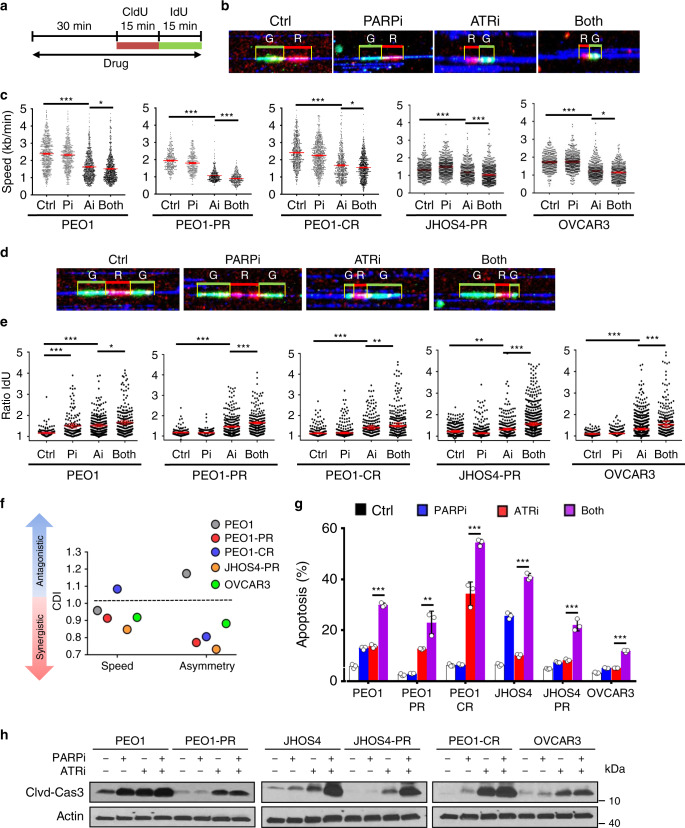
Combination PARPi–ATRi increases replication stress and apoptosis. **a**–**f** Experimental design for replication fork analysis for parental *BRCA*^MUT^ (PEO1), acquired PARPi and platinum-resistant (PEO1-PR, PEO1-CR, and JHOS4-PR), and platinum-resistant *CCNE1*^AMP^ (OVCAR3) cells were pretreated with PARPi (1 μM), ATRi (1 μM), or combination for 30 min, subsequently pulse-labeled with CldU (red) followed by IdU (green) for 15 min each, in the continuous presence of inhibitors. **b**, **c** Replication fork speed as calculated by length of track/duration both pulses and >100 intact unidirectional tracks were counted. ATRi exposure attenuated fork speed compared with control (*P* < 0.0001, all lines). PARPi–ATRi further reduced fork speed compared with ATRi alone in all lines (PEO1-PR and JHOS4-PR, *P* < 0.0001; PEO1-CR, *P* = 0.02; OVCAR3, *P* = 0.04; PEO1, *P* = 0.03). **d**, **e** Fork asymmetry as calculated by long green/short green length of replication initiation tracks and >100 intact initiation tracks were counted for each condition. PARPi treatment produced asymmetric forks in parental cells (PEO1, *P* < 0.0001). ATRi caused an increase in fork asymmetry in all lines (PEO1, PEO1-PR, PEO1-CR, and OVCAR3, *P* < 0.0001; JHOS4-PR, *P* = 0.0102). ATRi with PARPi further increased asymmetric fork ratios in all models (PEO1-PR, JHOS4-PR, and OVCAR3, *P* < 0.0001; PEO1-CR, *P* = 0.005; PEO1, *P* = 0.01). Data shown are median ± 95% CI*;* *n* = 2 biological independent samples and two slides per condition counted for DNA combing. One-way ANOVA followed by nonparametric Kruskal–Wallis test for (**c**) and (**e**). **f** The coefficient of drug interaction (CDI) was calculated to determine drug interaction effects. CDI < 1 indicated synergism, CDI = 1 additivity, CDI > 1 antagonism (**g**, **h**). Cells treated with control, PARPi (1 μM), ATRi (1 μM), or combination for 3 days (PEO1, PEO1-PR, PEO1**-**CR, and OVCAR3) and 5 days (JHOS4 and JHOS4-PR). Apoptosis was evaluated by Annexin V-APC and propidium iodide staining (**g**). Apoptosis was higher in PARPi–ATRi group compared with ATRi monotherapy in all cell lines (PEO1-PR, *P* = 0.0022; all remaining lines *P* < 0.001). Immunoblot detection of cleaved-caspase-3 in cells treated as indicated (**h**). Data shown mean ± SD (*n* = 3 biologically independent samples), individual samples are presented as data points. One-way ANOVA followed by Tukey’s multiple-comparisons test for (**g**). ***P < 0.001, **P < 0.01, *P < 0.05. Source data are provided as a source data file.

Since DSBs can occur as a consequence of collapsed replication forks, drug effects on replication fork progression was studied using DNA combing (Fig. 5a). Cells were incubated with drug (30 min), pulsed with CIdU (15 min) then IdU (15 min; Fig. 5a). Cells were evaluated for phosphorylated and total CHK1 at 30 min and at 1 h. Phospho-CHK1 levels decreased with ATRi treatment as early as 30 min in both parental, and acquired PARPi/platinum-resistant models (Supplementary Fig. 6). PARPi monotherapy did not significantly affect fork speed in acquired PARPi-resistant (PEO1-PR, JHSOS4-PR), platinum-resistant (PEO1-CR), *CCNE1*^AMP^ (OVCAR3), or parental cells (PEO1; *P* > 0.05 in all lines Fig. 5b, c). As expected, ATRi exposure alone caused a significant attenuation in fork speed (kb/min) compared with controls (PEO1, PEO1-PR, PEO1-CR, JHOS4-PR, OVCAR3, *P* < 0.0001, all lines; Fig. 5c). Significantly, combination PARPi–ATRi further reduced fork speed compared with ATRi alone in acquired PARPi-resistant cells (PEO1-PR, JHOS4-PR, *P* < 0.0001), platinum-resistant (PEO1-CR, *CCNE1*^AMP^ OVCAR3, *P* < 0.05) and parental cells (PEO1, *P* < 0.05; Fig. 5c).

Impaired replication may be an outcome of two possible scenarios: a result of reduced DNA polymerization rate or increased frequency of fork stalling or collapse. To distinguish between these two possibilities, we analyzed the fates of two forks emanating in opposite directions from the same origin (Fig. 5d, e). Decrease in the general polymerization speed would affect each direction equally (ratio close to 1) whereas fork stalling would affect them independently, resulting in fork asymmetry^58^. PARPi treatment did not produce asymmetric forks in acquired PARPi or platinum-resistant models (PEO1-PR, PEO1-CR, JHOS4-PR, and OVCAR3) as it did in parental cells (PEO1, *P* < 0.0001; Fig. 5e). ATRi exposure alone caused a significant increase in fork asymmetry in all models tested including acquired PARPi/platinum-resistant cells, including *BRCA2*^MUT^ parental cells (PEO1, PEO1-PR, PEO1-CR, JHOS4-PR, and OVCAR3, *P* < 0.0001 in all cell lines). Most importantly, the combination of ATRi with PARPi further increased asymmetric fork ratios in PARPi/platinum-resistant models (PEO1-PR, *P* < 0.0001; PEO1-CR, *P* = 0.0057; JHOS4-PR, *P* < 0.0001; OVCAR3, *P* < 0.0001) as well as parental cells (PEO1, *P* = 0.018; Fig. 5e). Importantly, combination of PARPi–ATRi treatment synergistically increased fork asymmetry in PARPi/platinum-resistant cells but not in sensitive cells (CDI = 0.7 PEO1-PR, CDI = 0.8 PEO1-CR, JHOSH4-PR CDI = 0.7, OVCAR3 CDI = 0.8; Fig. 5f). These results suggest that combination of PARPi–ATRi causes replication fork stalling and collapse in PARPi/platinum-resistant cells to a level that exceeds that achieved by ATRi monotherapy. In summary, PARPi–ATRi treatment exerts its dominant effects on replication by slowing fork progression and by increasing fork asymmetry that leads to DSBs.

### Combination PARP and ATR inhibition increases apoptosis

We next evaluated if the increase in replication stress and accumulation of DNA DSBs observed with PARPi–ATRi treatment precedes increased levels of apoptosis. PARPi monotherapy led to a significant increase in early and late apoptotic cells in parental *BRCA*^MUT^ cells (2.2-fold PEO1; 4-fold JHOS4; *P* < 0.001 for both lines), but had minimal effect in all PARPi/platinum-resistant lines (Fig. 5g, Supplementary Fig. 7). While ATRi monotherapy demonstrated a significant increase in apoptosis in PARPi/platinum-resistant *BRCA2*^MUT^ cells (5-fold vs control in both PEO1-PR, *P* < 0.001; PEO1-CR, *P* < 0.001), combination PARPi–ATRi led to a significantly higher increase in apoptosis [1.8-fold and 1.6-fold vs ATRi for PEO1-PR (*P* = 0.002) and PEO1-CR (*P*< 0.001), respectively].  In addition, in PARPi-resistant *BRCA1*^MUT^ and *CCNE1*^AMP^ cells, while ATRi monotherapy had minimal effect, combination PARP-ATRi significantly increased the percentage of apoptotic cells (JHOS4: 2.8-fold; JHOS4-PR: 2.2-fold; OVCAR3: 1.9-fold; *P* < 0.001 in all cell lines; Fig. 5g). These results further correlated with the increase in cleaved-caspase-3, a marker of apoptosis, observed with PARPi–ATRi more so than with ATRi alone in all resistant cell lines (PEO1-PR: 1.4-fold, *P* = 0.039; JHOS4-PR: 13.9-fold, *P* = 0.0069; PEO1-CR: 1.5-fold, *P* = 0.0169; OVCAR3: 1.5-fold, *P* = 0.0877; Fig. 5h). Taken together, PARPi–ATRi combination increases replication stress and causes DNA DSB in PARPi/platinum-resistant cells, ultimately leading to cell death via apoptosis.

### PARPi–ATRi causes tumor regression in drug-resistant PDXs

**Fig. 6:**
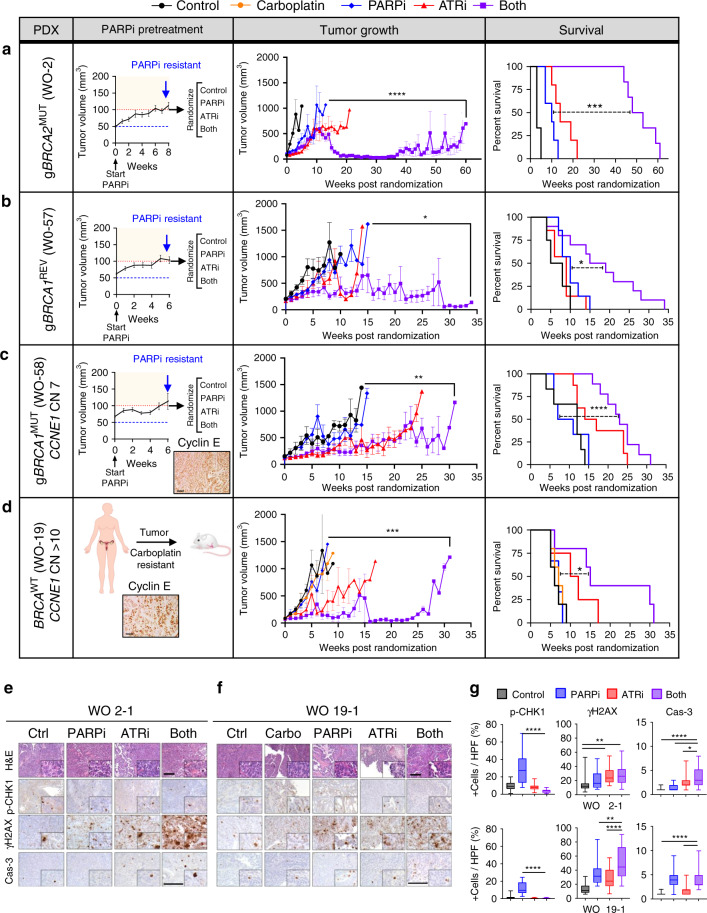
Combination effects of PARPi–ATRi in drug-resistant ovarian cancer PDXs. **a**–**d** PARPi pretreatment tumor growth curve (left), tumor volume growth curve (middle) and survival by Kaplan–Meier analysis (right) after randomization. **a** WO-2PR was from a g*BRCA2* mutation carrier whose PDXs were treated with olaparib until progression. Treatment groups: (1) control (*n* = 3), (2) PARPi 100 mg/kg/day OG 6 days weekly (*n* = 5), (3) ATRi 50 mg/kg/day OG 5 days weekly (*n* = 5), (4) PARPi 50+ATRi 50 mg/kg/day OG 5 days weekly (*n* = 6). Due to progression in combination group by week 10, PARPi was increased to 75 mg/kg/day 6 days weekly. **b** WO-57 was from a PARPi-resistant g*BRCA1* mutation carrier. Treatment groups: (1) control (*n* = 4), (2) PARPi 75 mg/kg/day OG 6 days weekly (*n* = 7), (3) ATRi 40 mg/kg/day OG 5 days weekly (*n* = 7), (4) Both (*n* = 10). **c** WO-58 was from a PARPi-resistant g*BRCA1* mutation carrier with elevated *CCNE1* copy number and protein. Treatment groups similar to (**b**): (1) control (*n* = 6), (2) PARPi (*n* = 6), (3) ATRi (*n* = 8), (4) Both (*n* = 9). **d** WO-19 was from a platinum-resistant, *BRCA*^WT^ patient with *CCNE1* amplification and protein overexpression by IHC magnification ×20. Scale bar = 50 μm. Treatment groups: (1) control (*n* = 5), (2) carboplatin 30 mg/kg IP weekly (*n* = 5), (3) PARPi 75 mg/kg/day OG 5 days weekly (*n* = 3), (4) ATRi 50 mg/kg/day OG 5 days weekly (*n* = 4), (5) Both (*n* = 6). Tumor growth shown is mean ± SEM. Longitudinal analysis by Linear Mixed-Effects modeling with type II ANOVA and pairwise comparisons across groups. Survival is shown by Kaplan–Meier curve using the Mantel-Cox log-rank test. *P* values provided in Supplementary Fig. 8. **e**–**g** H&E 10× and IHC 20× with 40× insets of pCHK1(S345), γH2AX, and cleaved-caspase-3 in WO-2PR and WO-19 PDXs at 2 weeks post-randomization. In box plots, bounds of boxes show interquartile range, whiskers show maximum and minimum, center lines indicate median. One-way ANOVA followed by Tukeys multiple comparison test for (**e**–**g**) (*n* = 27 HPF per group *n* = 3 mice, 9 HPF per tumor). ****P < 0.0001, WO-2 γH2AX *P* = 0.0039 (top), 0.0072 (bottom), cleaved-caspase-3 *P* = 0.0118, WO-19 γH2AX *P* = 0.0082. Scale bar = 100 μm. ****P < 0.0001, ***P < 0.001, **P < 0.01, *P < 0.05. Data are provided as a source data file.

We next tested whether the synergistic increases in replication stress and cell death caused by PARPi–ATRi would translate to increased efficacy in acquired PARPi-resistant tumors in vivo. Using the g*BRCA2*^MUT^ PDX model WO-2^42^, PARPi-resistant tumors were developed after >20 weeks of PARPi. PDX and parent tumors were evaluated using whole exome sequencing (~160× coverage) and bioinformatic analyses to identify tumor-specific alterations. The original germline *BRCA2* mutation (*BRCA2* E2906Gfs*12) was retained and no secondary mutations were found in the *BRCA2* allele, but a total of 22 new mutations were identified in this PARPi-resistant PDX. Mutation in *EXO1* (involved in DNA mismatch repair and HR)^55^ was found in this PDX as well as in *BRCA2*^*MUT*^ PEO1-PR cells (Supplementary Table 2). This model demonstrated RAD51 foci by immunohistochemistry with PARPi treatment suggesting HR restoration (Supplementary Fig. 11a). PARPi-resistant tumors were first treated with a PARPi until a 2-fold increase in volume) and then randomized to PARPi–ATRi treatment arms (Fig. 6a Left). Combination PARPi–ATRi resulted in near-complete tumor regression, increasing median overall survival (OS) > 16-fold, > 5-fold, and > 3-fold compared with control (*P* = 0.0011), PARPi (*P* = 0.0007), and ATRi alone (*P* = 0.0007) in the acquired PARPi-resistant *BRCA2*^MUT^ PDX (Fig. 6a, Supplementary Figs. 8a, 9a). While there was disease progression on all monotherapies, PARPi–ATRi caused complete regression (complete response, CR) by 25 weeks after randomization with ovarian sizes <30 mm^3^, non-palpable tumors in 50% (*n* = 3/6 mice) and therapy was stopped (Supplementary Figs. 8a, 9a). All mice had pretreatment with olaparib for 3–9 weeks before starting combination PARPi–ATRi. With regard to drug toxicity, three of six mice in the combination group required a dose reduction after treatment (10, 15, and 16 weeks) due to weight decrease >15%. Weight improved and stabilized after dose reduction (Supplementary Fig. 10a). The three PDXs that continued at the full combination dose had complete responses and the mice treated at the reduced dose had partial responses (Supplementary Fig. 9a) suggesting a narrow dose–response range for maximal efficacy. About 33% of mice survived until 46 and 59 weeks and were ultimately sacrificed for old age and to look for evidence of residual disease; each found to have low-volume disease (100–300 mm^3^).

To evaluate if PARPi–ATRi was effective in a PARPi-resistant, g*BRCA1*^MUT^ setting, the WO-57 PDX model was developed from a patient who responded but then progressed on PARPi (rucaparib) (Fig. 6b). WES revealed a *BRCA1*^REV^ mutation in the patient and PDX as a result of frame shift in the coding sequence of the gene (*BRCA1* H839Qfs*12) that brought the original germline frameshift alteration (*BRCA1* I815Ffs*31) back in frame (Supplementary Table 2). After PARPi resistance was verified with a 2-fold tumor volume increase (Fig. 6b, left), the addition of ATRi (PARPi–ATRi combination) led to an increase in tumor regression relative to PARPi (*P* = 0.0452) and ATRi (*P* = 0.0104) and improved median overall survival of 2.6-fold, 1.7-fold, 1.9-fold compared with control (*P* = 0.0118), PARPi (*P* = 0.0292), and ATRi (*P* = 0.0078), respectively (Fig. 6b, Supplementary Figs. 8b, 9b). While there was disease progression in all the monotherapy groups by 6 weeks of treatment, there was a 40% (4/10 mice) partial response rate in the PARPi–ATRi group and synergy index of 0.80 indicating a synergistic effect with combination treatment. PAPRi-ATRi at this lower dose level (no dose reductions required) was durable with mice on treatment up to 34 weeks and tolerable with mice maintaining overall stable weights (Fig. 6b, Supplementary Fig. 10b).

Lastly, PAPRi-ATRi was evaluated in two *CCNE1*^AMP^ models. WO-58 PDX was derived from a g*BRCA1* mutant carrier whose tumor progressed on olaparib. Her tumor showed the similar *BRCA1* 5370C>T (R1751X) alteration but notably also showed a *CCNE1* copy number of 7 by WES (Supplementary Table 2). Cyclin E protein was highly expressed confirmed by IHC (Fig. 6c). Combination PARPi–ATRi was synergistic in decreasing colony formation in primary cell lines derived from this tumor (Supplementary Fig. 11b). After PDX tumors were transplanted, selected for PARPi resistance, randomized, PAPRi-ATRi treatment showed increased OS compared with PARPi alone (*P* < 0.0001; Fig. 6c, Supplementary Figs. 8c, 9c). Combination increased tumor regression 3.2-fold with a 12.4% partial response rate with ATRi alone versus a 40% partial response rate in the combination group (ATRi vs both *P* = 0.0320). Combination treatment was also synergistic with a synergy index of 0.89. Treatment was well-tolerated and no dose reductions were required. At least half of the mice in the combination group were on treatment for more than 23 versus 9 weeks for PARPi group (Supplementary Fig. 10c).

WO-19 PDX was derived from a platinum-resistant HGSOC with *CCNE1* copy number of >10. Resistance to carboplatin and PARPi was demonstrated with tumors reaching >1000 mm^3^ within 5 weeks, similar to control (*P* > 0.05; Fig. 6d). Combination PARPi–ATRi led to increased tumor regression relative to PARPi monotherapy (*P* = 0.0003) and increased median OS of 2.5-fold, 2.1-fold, and 2.1-fold compared with control (*P* = 0.0142), carboplatin (*P* = 0.0206), and PARPi (*P* = 0.0462; Fig. 6d, Supplementary Fig. 8d, 9d). Although there was not a statistically significant difference between PARPi–ATRi and ATRi monotherapy, there was an increase in median overall survival (OS) of 4 weeks with PARPi–ATRi treatment (median OS: Combination 15 vs 11 weeks with ATRi, *P* > 0.05). Further, while there was disease progression in all the monotherapy groups within the first 3–4 weeks of treatment, there was a 40% (*n* = 2/5 mice) complete response rate in the combination PARPi–ATRi group (Fig. 6d, Supplementary Figs. 8d, 9d). Further, combination treatment was synergistic with a synergy index of 0.94. With regard to drug toxicity, none of the mice required a dose reduction and mice bearing PDXs maintained their weight throughout the study duration of 33 weeks (Supplementary Fig. 10d). In summary, PDX experiments demonstrate that PAPRi-ATRi induces tumor regression and is tolerable for sustained treatment time leading to durable responses.

Drug effects on tumors were evaluated by measuring pCHK1, γH2AX, and cleaved-caspase-3 before and after 2 weeks of treatment in WO-2 and WO-19 PDX models (Fig. 6e–g). Consistent with in vitro observations, PARPi induced pCHK1 and the addition of ATRi abrogated this increase (Fig. 6e–g). Furthermore, γH2AX and cleaved-caspase-3 increased with combination PARPi–ATRi compared with monotherapy. Thus, drugs recapitulated in vitro findings, indicating that this drug combination targets expected ATR pathway effectors in PDX models.

## Discussion

Herein, we developed PARPi and platinum-resistant in vitro, and in vivo models from germline *BRCA*1/2 mutant patient tumors after prolonged treatment (>1 yr) with drug (defined as acquired resistant) to emulate how patients are treated in the clinic. With emergence of resistance, these in vitro and in vivo models developed multiple new mutations, often in DNA repair genes, as well as chromosomal rearrangements and copy-number alterations. Also, overexpression of drug efflux pumps such as MDR1 was identified (Fig. 1, Supplementary Fig. 2, Supplementary Tables 1, 2). The acquisition of PARPi/platinum resistance and *CCNE1* amplification associated high levels of replication stress, is accompanied by increased baseline activation of ATR/CHK1, suggesting sensitivity to ATRi (Fig. 1d). However, ATRi monotherapy was less effective than PARPi–ATRi, which synergistically decreased cell viability and colony formation in diverse acquired and de novo PARPi and platinum-resistant models (including *CCNE1*^AMP^). This sensitivity was observed using doses with minimal off-target effects^41^ (Fig. 2). Consistent with these observations, OVKATE, which is not *CCNE1* amplified, has lower levels of replication stress as demonstrated by lower pCHK1 and pATR and is not sensitive to the PARPi–ATRi combination (Fig. 2). Notably, we showed combination PARPi–ATRi leads to tumor regression and a significant increase in overall survival in clinically relevant HGSOCs, including *BRCA2*^MUT^ and *BRCA1*^MUT^ with acquired resistance to PARPi, and *BRCA* wild-type, *CCNE1*^AMP^ platinum-resistant PDX models. Dosing used in PDX experiments are comparable to that in the clinic for humans. PDX experiments demonstrate that combination PARPi–ATRi is well-tolerated for sustained treatment durations (>50 weeks) to where mice were ultimately often euthanized secondary to old age. We show that this combination induces tumor regression that is durable not only in acquired PAPRi-resistant but platinum-resistant PDX models with *CCNE1* amplification overcoming multiple mechanism of resistance. Indeed, synergy between ATRi and PARPi has been reported in HR-deficient HGSOC in vivo^40^ or HR-proficient genetic contexts in vitro^57^ further supporting our conclusions. Likewise, we and others showed that targeting downstream of ATR with a CHK1/2 or WEE1 inhibitor synergizes with PARPi in *BRCA*^MUT^ and *BRCA* wild-type models^31,59,60^.

Our data suggest combination PARPi–ATRi is synergistic in many models of PARPi and platinum resistance yet the driving mechanism of drug synergy may differ. As we previously published and expand further in this current study, the mechanism of synergy in the PARPi-sensitive *BRCA*^MUT^ setting, results from combination treatment effects on cell-cycle regulation, and replication fork stability^40^. Specifically, we showed that combination PARPi–ATRi treatment inhibits PARPi-mediated ATR/CHK1 signaling pathway activation and G2-M arrest, leading to increased replication fork collapse, as determined in the current study by both γH2AX in S phase and replication fork asymmetry.

In contrast to PARPi-sensitive cell lines which lack accumulation for RAD51 due to *BRCA1/2* mutation, the sensitivity of acquired and de novo PARPi-resistant and carboplatin-resistant models to the PARPi–ATRi combination was often correlated with the ability of ATRi to suppress RAD51 accumulation (Fig. 4). Restoration of RAD51 nuclear foci has been described as a biomarker for functional HR and PARPi resistance^52,55^. This correlation was observed in several acquired and de novo PARPi and platinum-resistant cell lines (PEO1-PR, UWB/*BRCA1*^+/−^, PEO1-CR, and PEO4) and is similar to other reports showing ATRi induces an HR-deficient state in PARPi-resistant cells with ectopic BRCA1 expression^53^. In these lines, PARPi–ATRi decreased replication fork stability and increased cell death compared with ATRi alone (Figs. 4 and 5). As expected, the effect of PARPi on G2 build up in BRCA1/2-deficient lines was not observed in these PARPi-resistant lines, as ATRi-driven blockade in RAD51 accumulation was tied to concomitant loss of G2-M checkpoint control (Fig. 3). Of note, PARPi and platinum-resistant lines restored RAD51 accumulation independent of *BRCA* reversion mutations (Fig. 4), which puts in question the importance *BRCA1/2* sequencing in predicting the acquisition of resistance. As cell lines and PDX models with acquired resistance developed many new mutations after prolonged treatment with PARPi, there can be various combinations of these mutations that may account for PARPi–ATRi drug synergy (Fig. 1, Supplementary Tables 1 and 2). In summary, for these models where RAD51 accumulation is restored, our data is consistent with the model that PARPi causes reliance on ATR for fork stabilization, and ATRi causes reliance on PARPi by suppressing RAD51 accumulation.

Importantly, however, not all acquired and de novo PARPi-resistant models in our study exhibited the restoration of RAD51 accumulation following DNA damage. Specifically, we show that the *BRCA1*^MUT^ JHOS4-PR or *BRCA2*^MUT^ Kuramochi cells did not exhibit RAD51 foci upon treatment with PARPi, but were PARPi-resistant (Figs. 2 and 4). Thus, our studies demonstrate that RAD51 foci does not always predict sensitivity to PARPi, which is a new and clinically relevant finding. The means of PARPi resistance in these cell lines is not clear, however, it is important to note that *XPC* amplification was observed in JHOS4-PR line and knockdown of *XPC* in this line increased PARPi sensitivity (Supplementary Table 1, Supplementary Fig. 2c). Even though RAD51 accumulation was not restored in these acquired and de novo PARPi-resistant lines, combination PARPi–ATRi treatment resulted in increased γH2AX, replication fork asymmetry (JHOS4-PR), and a synergistic increase in cell killing (Figs. 4 and 5). In these cells, the means of increased reliance on either ATRi or PARPi is not clear. However, of note, ATR has been observed to cause a number of effects on replication fork dynamics, and PARP is a key responder to defects in Okazaki fragment ligation^33,61^. It is conceivable that PARPi and ATRi synergize in these cells through a complete novel feed-forward mechanism of dual dependency at replication forks, independent of PARP’s roles in base excision repair and ATR’s function in promoting RAD51 recruitment.

Lastly, we show that combination PARPi–ATRi was also active in platinum-resistant *CCNE1*^AMP^ cells and PDX models in a manner that may involve many of the mechanisms described above. However, in addition to these mechanisms, CCNE1-overexpression has been reported to cause premature S phase entry, leading to increased levels of replication stress^49,50^. Indeed, this increase in replication stress was observed in our studies as well, as evidenced by higher levels of pCHK1 and total CHK1 in *CCNE1-* amplified cells (Fig. 1d). Consistent with our interpretation of multifactorial mechanisms of synergy, ATRi disrupted PARPi-induced RAD51 accumulation similar to some of the PARPi-resistant *BRCA*^MUT^ models described above. However, unlike these models, combination treatment disrupted the G2-M cell-cycle regulation of the G2 build up in the *CCNE1*^AMP^ cells (Fig. 3), indicating distinct mechanisms of genome destabilization. Combination also increased replication fork instability as determined by both γH2AX in S phase and replication fork slowing and asymmetry resulting in apoptosis (Figs. 4 and 5). These results indicate that combination PARPi–ATRi creates multiple genome-destabilizing vulnerabilities that are exacerbated by CCNE1-associated replication stress. Importantly, our findings expand the utility of the PARPi–ATRi combination to include *CCNE1*^AMP^ HGSOCs.

In summary, we demonstrate PARPi–ATRi drug synergy in a comprehensive panel of acquired and de novo PARPi-resistance and platinum-resistant *CCNE1*^AMP^ in vitro and PDX models. We have shown that regardless of the PARPi and platinum-resistance mechanisms in operation (including reacquisition of HR through *BRCA* reversions), the combination PARPi–ATRi leads to increased DNA damage and durable ovarian tumor regression with a dramatic increase in overall survival in acquired and de novo PARPi and platinum-resistant *BRCA* mutant and *CCNE1*^AMP^ PDX models. Tolerability of long-term treatment (e.g., >50 weeks) with PARPi combinations, as we have demonstrated with PARPi–ATRi, will be critical for PARPi combination therapies to move forward in the clinic. These studies provided preclinical in vitro and in vivo data to support moving combination PARPi–ATRi forward into the clinic, and have led to the trial design for the Combination ATR with PARP Inhibition or the CAPRI trial which will be evaluating AZD6738 and olaparib for recurrent ovarian cancer.

## Methods

### Cell lines

PEO1 (*BRCA2*^MUT^), Kuramochi (*BRCA2*^*MUT*^), OVKATE (*BRCA*^*WT*^ and *CCNE1* copy normal), FUOV1 (*CCNE1* amplified; *CCNE1*^AMP^), COV318 (*CCNE1*^AMP^), and OVCAR3 (*CCNE1*^AMP^) ovarian cancer cells were grown in RPMI media with 10% FBS and penicillin/streptomycin. PEO1 and PEO4 lines were a generous gift from Dr. Andrew Godwin, University of Kansas, Kansas City, KS. JHOS4 (*BRCA1*^MUT^) ovarian cancer cells were grown in DMEM/F12 media with 10% FBS, and penicillin/streptomycin. PARPi-resistant cell lines (PEO1-PR, PEO1-PR1, PEO1-PR2, JHOS4-PR, JHOS4-PR1, and JHOS4-PR2) and carboplatin-resistant cell lines (PEO1-CR, PEO1-CR1, and PEO1-CR2) were developed by long-term drug exposure (>12 months; 0.5–3 µM of PARPi, 0.1–1 µg/ml of carboplatin). Authenticity was confirmed by short tandem repeats by the Wistar Genomics Core (Wistar Institute, Philadelphia, PA). Cell lines were mycoplasma negative.

### Knockout of 53BP1 and knockdown of XPC

To develop a 53BP1-knockout line, UWB1.289 cells were engineered to stably express Cas9 using LentiV_Cas9_puro (Addgene, 108100). sgRNA expressing vector LRG2.1 (Addgene, 108098) was modified to replace GFP with blasticidine (LR2.1b). 53BP1 sgRNA (5′ GTTGACTCTGCCTGATTGTA 3′) was expressed in the UWB_Cas9 lines using the LRG21.1b vector. After selection with blasticidine, sgRNA-transduced cells were plated and individual colonies were isolated. Clones with complete depletion of 53BP1 were pooled for further experiments. UWB1.289 parent, with *BRCA1*^+/−^ and *53BP1*^−/−^, were maintained in DMEM/F12 supplemented with 5% fetal bovine serum, 5 μg/mL insulin, 10 ng/mL hEGF, 0.5 mg/mL hydrocortisone, 100 ng/mL cholera toxin, and 1x penicillin/streptomycin. The UWB.289 *BRCA1*^+/−^ cells were maintained in 200 μg/mL G-418 to select for the BRCA1 expressing cells.

JHOS4-PR cells were transfected were transfected with 20 nM of siRNA smartpool targeting XPC (#L-016040-00-0005, Horizon Discovery) or non-targeting control siRNAs (#D-001810-01-05, Horizon Discovery) using lipofectamine RNAiMAX, according to the manufacturer’s guidelines. After cells were treated with Olaparib, survival was assessed by MTT. The XPC knockdown was confirmed by western blot.

### In vitro survival, colony formation, cell cycle, immunofluorescence, and apoptosis

Cells were seeded and treated with the indicated doses of carboplatin (Hospira Inc., King of Prussia, PA), PARPi (AZD2281, AstraZeneca, Wilmington, DE), ATRi (AZD6738, AstraZeneca, Wilmington, DE) or both. Cell survival and colony formation ability were performed to evaluate the efficacy of PARPi and ATRi at indicated doses by MTT (at 5 days) and crystal violet staining (at 10 days), respectively. For cell-cycle analysis and apoptosis detection by flow cytometry and immunofluorescence (IF) staining, cells were plated and then treated with 1–2 μM PARPi, 1 μM ATRi, or both for indicated times.

For cell cycle, cells were incubated with drugs (24 h) and bromodeoxyuridine (BrdU; 10 µM) was added for 2 h before harvest. Cells were labeled with FITC-conjugated anti-BrdU and propidium iodide (PI) solution and analyzed by flow cytometry (BD FACSCalibur, BD Biosciences, San Jose, CA). Data were analyzed by FlowJo software (Tree Star, Inc., Ashland, OR).

For IF, cells were seeded onto a coverslip, treated for 24 h, fixed and stained with RAD51 and Geminin antibody (Supplementary Table 3) and images were quantitated for geminin and RAD51 positive cells (>5 RAD51 foci per cell) using ImageJ software (NIH, Bethesda, MD).

For apoptosis, cells were incubated with drug for 3–5 days and evaluated using an Annexin V Flow Kit (BD Biosciences, Franklin Lakes, NJ) according to the manufacturer’s instructions. The data were analyzed by FlowJo software (Tree Star, Inc., Ashland, OR). Apoptosis was also evaluated by cleaved-caspase-3 (Supplementary Table 3).

### Western blot

Cells and tissues were harvested and lysed in Laemmli sample buffer (BioRad, Hercules, CA). Cell lysates were separated on SDS-PAGE gels and immunoblotted with primary antibodies (Supplementary Table 3). Band intensity was quantified using ImageJ software (NIH, Bethesda, MD).

### DNA combing assay

Replication fork speed and asymmetry was evaluated using the molecular combing assay. Exponentially growing cells were pretreated with DMSO, PARPi (AZD2281; 1 μM), ATRi (AZD6738; 1 μM), or the combination for 30 min. Cells were subsequently pulse-labeled with 100 μM 5-chloro-2′-deoxyuridine (CldU; cat. # C6891, Sigma-Aldrich, St. Louis, MO) followed by 100 μM 5-iodo-2′-deoxyuridine (IdU; cat. # I7125, Sigma-Aldrich, St. Louis, MO) for 15 min each, in the continuous presence of inhibitors. Afterward, cells were harvested and embedded into agarose plugs using the Genomic Vision FiberPrep® kit (Genomic Vision, Bagneux, France). DNA extraction, combing and immunostaining was performed according to the EasyComb service procedures (Genomic Vision, Bagneux, France). Coverslips were scanned with FiberVision® scanner and images were analyzed through Genomic Vision FiberStudio® software (Genomic Vision, Bagneux, France). Intact CldU (red) and IdU (green) replication tracks flanked by counterstaining were selected and reviewed for further validation.

#### PDX studies and immunohistochemistry

NSG mice (NOD/SCID IL2Rγ^−/−^) were purchased from the Stem Cell and Xenograft Core at the University of Pennsylvania (UPENN). All mice were housed according to the Institutional Animal Care and Use Committee at UPENN (temperatures of 68–79 F with 40–60% humidity, 12-h light/12-h dark cycle). Tumor was obtained from ovarian cancer debulking surgeries conducted at the Hospital of the UPENN and Pennsylvania Hospital (IRB# 702679). PDXs were generated by surgically engrafting 3–4 pieces (2–3 mm^3^ each) orthotopically to the mouse fallopian tube/ovary^40,62^. For preclinical trials, cryopreserved tissue was thawed and transplanted. All tumors generated from *BRCA*^*MUT*^ patients were pretreated with olaparib, and after a 2-fold increase in volume (confirming PARPi resistance), were randomized to treatment arms. Tumor volume was calculated by ultrasound (SonoSite Edge II Ultrasound System) and body weight was measured weekly. Tissue samples were fixed in formalin and embedded in paraffin for immunohistochemistry (IHC)^62^. The detection of cleaved-caspase-3, pCHK1, and γH2AX was performed as described in the supplementary methods (Supplementary Table 3). Weekly ultrasound and weight measurements were obtained for treatment groups in a blinded manner. Histology was scored by pathologists in a blinded manner.

### Next-generation sequencing and mutation analysis

DNA samples were extracted and fragmented using the truXTRAC™ FFPE DNA Kit (Covaris, Valencia, CA). Fragmented genomic DNA from patient tumors and normal tissues, PDX tumors, and cell line samples were used for Illumina TruSeq library construction (Illumina, San Diego, CA). Exonic regions of tumor, normal and PDX samples were captured in solution using the Agilent SureSelect v.4 kit (Agilent, Santa Clara, CA). Paired-end sequencing resulting in 100 bases from each end of the fragments for exome libraries and 125 bases for whole genome libraries was performed using Illumina HiSeq 2000/2500 instrumentation (Illumina, San Diego, CA).

Somatic mutations in tumor, normal and PDX samples were identified using VariantDx (PGDx, Baltimore, MD) custom software for identifying mutations in matched tumor and normal samples^63^. Sequence reads were aligned against the human reference genome (v.hg19) using ELAND (version 1.8.2; Illumina Inc., San Diego, CA). Potential somatic mutations and copy-number alterations excluding mouse-specific variants were identified using VariantDx custom software. We identified candidate mutations that were altered in >10% of distinct reads.

Cell lines were analyzed using whole genome sequencing. Sequence reads were aligned against the hg19 human reference genome using ELAND. Candidate somatic mutations in the protein coding regions of the exome, consisting of point mutations, insertions, and deletions were identified using Strelka v2.9.0 and Manta v1.3.1. To detect mutations that were likely to be somatic, we excluded mutations that appeared in <10% of the distinct reads and mutations tagged as COMMON in dbSNP VCF files. Candidate structural variants including deletions, linked amplicons, intra- and inter-chromasomal rearrangements were identified using Trellis v1.0 from whole genome sequenced cell lines^64^.

### RPPA analysis

The cells were collected and samples were lysed with RIFA buffer and analyzed using a reverse phase protein array (RPPA) platform at the MD Anderson Center RPPA core facility^65^. Antibodies targeting >300 proteins were included in this assay (see for detailed methods and antibody descriptions: http://www.mdanderson.org/education-and-research/resources-for-professionals/scientific-resources/core-facilities-and-services/functional-proteomics-rppa-core/index.html). The results were reported with normalized data at both linear and Log_2_ version. The relative protein expression levels were analyzed and presented in a heatmap.

### Statistical analyses

MTT, colony formation assays, flow cytometry, and western blots were performed using at least three biological replicates per sample and as three independent experiments. One-way ANOVA followed by Tukey’s post hoc comparison was performed for multiple group in vitro comparisons. Drug interaction between ATRi and PARPi, was analyzed using the coefficient of drug interaction (CDI) calculated for in vitro studies^40,66^. CDI = AB/(A × B); AB is ratio of two-drug combination group to control, and A or B is ratio of single drug to the control. CDI < 1 indicates synergism, CDI < 0.7 indicates significant synergism. Microsoft Excel (version 2016) was used for data collection and management. GraphPad Prism (GraphPad Software 8.4.2, San Diego, CA) was used for statistical analyses. For statistical power for in vivo studies, we transplanted 12 mice per arm based on prior experience^40^. After randomization, once pre-specified tumor volume was achieved, there were ~8 mice per arm (range 4–10). Randomization was performed using Tumor Manager software (v 3.3.4) by Biopticon. Mice that died for unknown reason (low tumor burden, normal weight, and condition scores) were excluded from analysis. Weekly ultrasound measurements, weights, and condition scores were obtained in a blinded manner. Longitudinal analysis of tumor growth was carried out by linear mixed-effect modeling on log pre-processed tumor sizes using the TumGrowth web tool (https://kroemerlab.shinyapps.io/TumGrowth/)^67^. Log transformed tumor volume was used to better satisfy normal distribution. Survival data were analyzed by Mantel-Cox log-rank test. Using the slopes obtained from the TumGrowth web tool^67^ as effects measurements, synergy under the Bliss definition of independence was used. The index defined as the ratio of sums of slopes (Both and Control over ATRi and PARPi), would indicates synergy, additivity, and antagonism if <1, 1, >1, respectively.

### Reporting summary

Further information on research design is available in the Nature Research Reporting Summary linked to this article.

## Supplementary information

Supplementary Information

Reporting Summary

## Data Availability

All whole genome and exome sequencing data generated for this study have been deposited in the NCBI GEO database (https://www.ncbi.nlm.nih.gov/gds/) under accession # PRJNA626435 and PRJNA626436. All the other data supporting the findings of this study are available within the article and its supplementary information files and from the corresponding author upon reasonable request. Source data are provided with this paper.

## Source data

Source Data
