# Supplementary material for: Combining PARP with ATR inhibition overcomes PARP inhibitor and platinum resistance in ovarian cancer models
Source: Nat Commun. 2020 Jul 24;11:3726. doi: 10.1038/s41467-020-17127-2 (PMC7381609; doi:10.1038/s41467-020-17127-2)
Supplement: Supplementary file 1 — Supplementary Information [file 41467_2020_17127_MOESM1_ESM.pdf]

Combining PARP with ATR inhibition overcomes PARP inhibitor and platinum resistance in ovarian cancer models

Kim, H et al.

Supplementary Figure 1

a

ID	Genetic Alteration	IC$_{50}$ PARPi (µM)	IC$_{50}$ Carbo (µg/mL)	IC$_{50}$ ATRi (µM)
PEO1	BRCA2^{MUT}	0.38	0.59	0.27
JHOS4	BRCA1^{MUT}	2.25	2.80	1.88
UWB	BRCA1^{MUT}	0.64	0.94	0.48
PEO1-PR	BRCA2^{MUT}	5.28	0.70	0.15
PEO1-PR1	BRCA2^{MUT}	4.34	1.08	
JHOS4-PR	BRCA1^{MUT}	18.54	2.12	0.81
KURAMOCHI	BRCA2^{MUT}	12.02	0.76	1.75
UWB/B1	BRCA1$^{+/-}$	50.08	19.78	1.36
UWB/53BP1	BRCA1^{MUT}	2.38	0.47	0.16
PEO1-CR	BRCA2^{MUT}	28.98	2.69	0.22
PEO1-CR2	BRCA2^{MUT}	3.2	6.26	
PEO4	BRCA2^{REV}	3.30	7.54	0.50
OVCAR3	CCNE^{AMP}	17.40	2.71	1.08
FUOV1	CCNE^{AMP}	4.50	18.50	0.60
COV318	CCNE^{AMP}	9.63	13.58	0.29
OVCATE	CCNE^{AMP}	6.07	21.30	2.30

b **c**

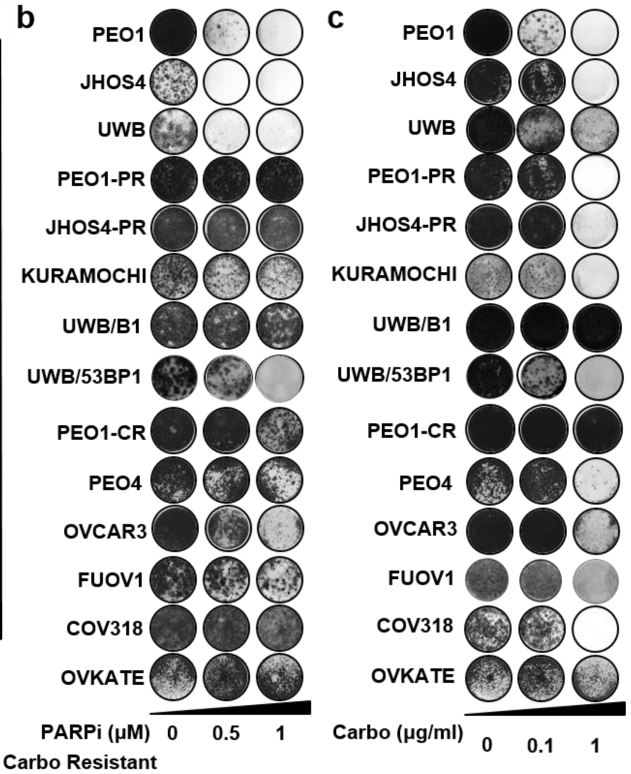

d

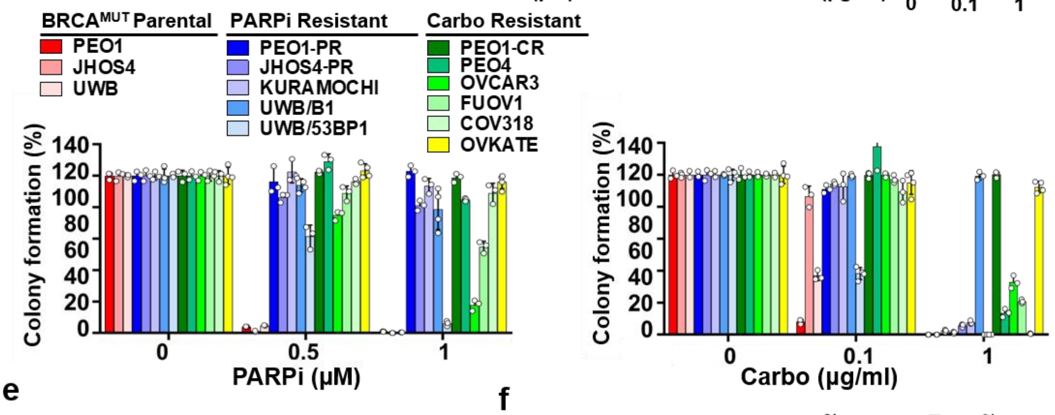

e

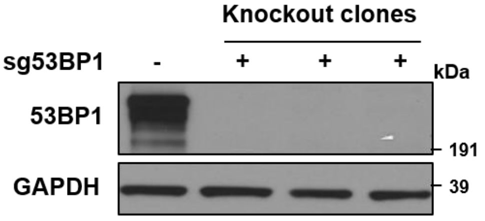

f

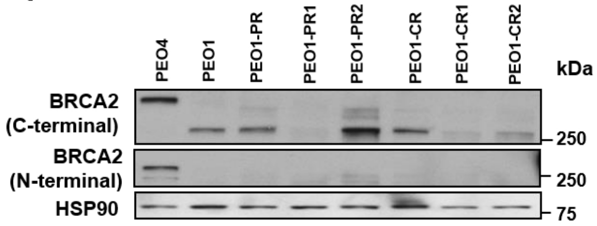

Supplementary Figure 1. Effects of PARPi and platinum treatment on survival, colony formation and ATR/CHK1 signaling. **(a)** IC_{50} after parental *BRCA*^{MUT}, PARPi-resistant and platinum-resistant cells PAPRi (0.1-10μM), carboplatin (0.1-10μg/ml), and ATRi (0.1-10μM) treatment after 5 days by MTT. Nonlinear regression curve was generated using MTT data (dose-response inhibition). IC_{50} was calculated by Graph Pad Prism. Data shown as mean±SD of a single representative experiment (n=3 independent biological samples). **(b-d)** Colony formation (CF) ability after treatment with increasing concentrations of **(b)** PARPi or **(c)** carboplatin at dose indicated in parental BRCA^{MUT} (PEO1, *BRCA2*^{MUT}; JHOS4, UWB *BRCA1*^{MUT}), PARPi-resistant lines (PEO1-PR; JHOS4-PR, Kuramochi, UWB/B1 where B1 denotes BRCA1+/-), and platinum resistant cell lines (PEO1-CR; PEO4; *CCNE1*^{Amp} OVCAR3, FOUV1, COV318) for 14 days. Cells were seeded with cell number normalized based on cell doubling time. **(d)** Quantification of CF with increasing concentrations of PARPi or carboplatin was performed using ImageJ. With PARPi treatment, 0.5μM, CF ability was higher in PARPi-resistant cells compared to parental lines (PEO1-PR vs PEO1, JHOS4-PR vs JHOS4, UWB/B1 vs UWB, UWB/53BP1 vs UWB, Kuramochi vs PEO1, P<0.0001). At PARPi 1μM: PEO1-PR vs PEO1, JHOS4-PR vs JHOS4, UWB/B1 vs UWB, Kuramochi vs PEO1, *P*<0.0001; but UWB/53BP1 vs UWB=0.1331). Treatment with Carboplatin 0.1μM, CF ability was higher in platinum-resistant cells compared to platinum-sensitive PEO1 for all lines (PEO1-CR, PEO4, OVCAR3, FUOV1, Cov318, OVKATE; P<0.0001). At Carboplatin 1μg/ml: PEO1-CR, OVCAR3, FUOV1, OVKATE, vs PEO1 *P*<0.0001; except PEO4, P=0.03; Cov318, P=0.99. One-way ANOVA analysis followed by Tukey's multiple-comparisons test was used and data shown as mean±SD (n=3 independent biological samples). **(e)** UWB and UWB 53BP1-/- clones were evaluated for 53BP1 protein by immunoblot. **(f)** Expression of functional BRCA2 protein was examined in PEO1 (*BRCA2*^{MUT}) cells and PARPi and platinum-resistant cells (PEO1-PR, PEO1-CR) including 2 clonal cell lines in each resistant cells (PEO1-PR1, PEO1-PR2, PEO1-CR1, PEO1-CR2). Nuclear extractions were analyzed by western blot for the BRCA2 proteins. PEO4 (*BRCA2* reversion mutation) was used as a positive control.

HSP90 used as loading control. Representative data of 3 biological repeats are shown. Source data are provided as a source data file.

Supplementary Figure 2

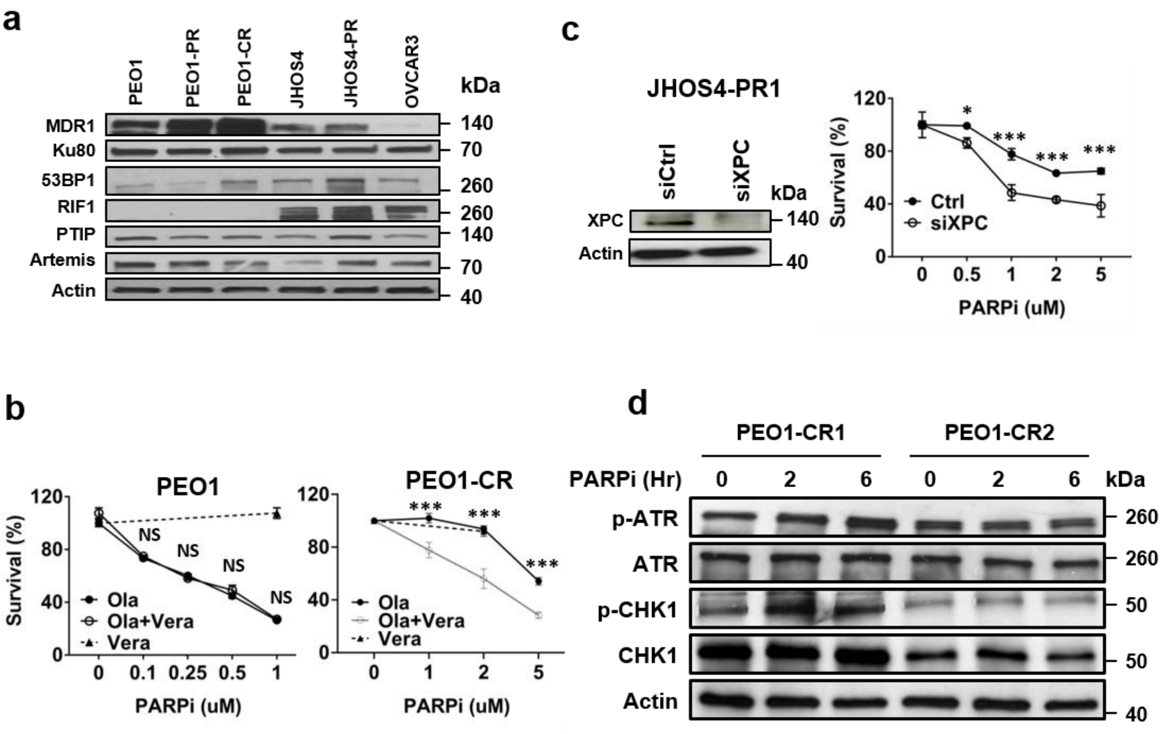

Supplementary Figure 2. Evaluation of resistance pathways in PARPi and platinum resistant cells **(a)** *BRCA1/2*^{MUT} cells (PEO1, JHOS4), PARPi-resistant cells (PEO1-PR, JHOS4-PR), and platinum-resistant cells (PEO1-CR, OVCAR3) were collected and western blot was performed for the indicated proteins. Representative data of 3 biological repeats are shown. **(b)** MTT assay shows PEO1 and PEO1-CR cell viability after olaparib, verapamil (MDR1 inhibitor at 1-2 µM) or both treatments at indicated doses for 5days. Olaparib+Verapamil vs Olaparib in 1-5µM PARPi dose, P<0.0001. **(c)** JHOS4-PR cells were transfected with siRNA to XPC and then treated with olaparib in a dose dependent manner and evaluated for % survival after 5 days by MTT. XPC immunoblot after knock down with siRNA to XPC. XPC-siRNA vs Control at 0.5µM PARPi, P=0.0244; 1 and 5 µM PARPi, P<0.0001. Two-way ANOVA was performed with Bonferroni post-test adjustment to calculate p values. Data are presented as the mean±SD; n=3 independent biological samples. **P*<0.05, ****P*<0.0001, and NS=non-significant. **(d)** PEO1-CR1 and PEO1-CR2 clones derived after

prolonged culture in olaparib 1-2μM were probed for phospo and total CHK1 and ATR after 0, 2, 6 hrs of olaparib treatment (1μM) by immunoblot. Representative data of 3 biological repeats are shown. Source data are provided as a source data file.

Supplementary Figure 3

a

Pathway	Proteins (up-regulated)	Proteins (down-regulated)
**PI3K-Akt**	S6_pS240_S244, S6_pS235_S236, S6, Akt_pS473, Akt_pT308, Akt, PI3K-p110-b, GSK-3a-b	PTEN, eIF4E_pS209, IGF1R_pY1135_Y1136, 4E-BP1, PDK1, IGFRb, AMPKa, p70-S6K1,
**ATR-CHK1**	ATR_pS428,	Wee1_pS642, Chk1_pS296,
**Energy Metabolism**	Transglutaminase, SDHA, SCD,	ACC1, FASN, LDHA, Glutaminase
**DNA repair, PARPi resistance**	BRD4, Rad51, BAP1, XPA	

b

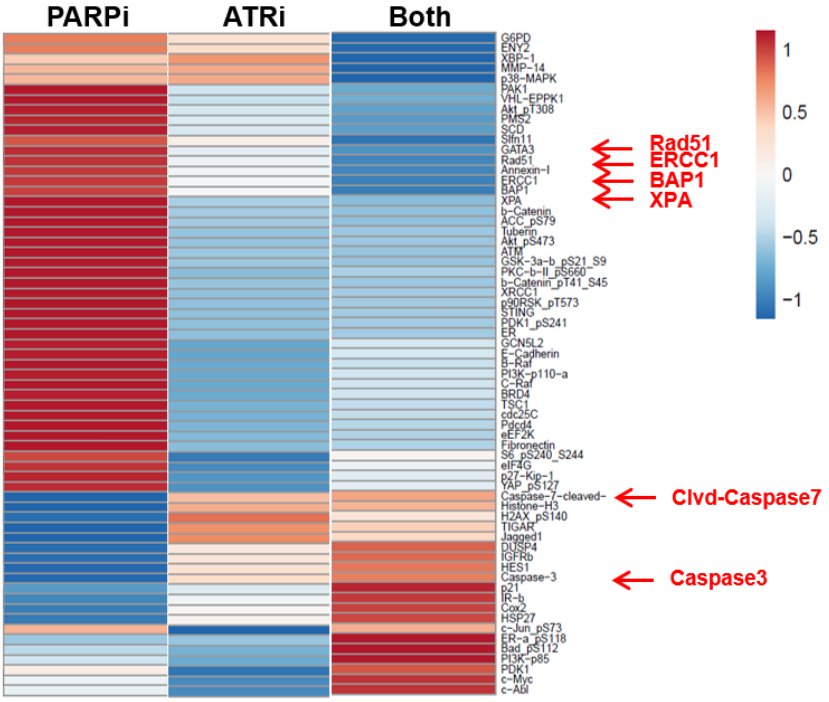

Supplementary Figure 3. RPPA analysis of acquired PARPi-resistant cells. (a) Protein alteration was analyzed by RPPA analysis. PEO1 PARPi resistant (PEO1-PR) cells were compared with PEO1 parental cells. Protein changes in top selected 4 pathways were examined. **(b)** PEO1-PR cells were treated with PARPi (1uM), ATRi (1uM), and both drugs for 24 hr then analyzed by RPPA. Proteins involved in DNA repair pathway and apoptosis were marked in red. Source data are provided as a source data file.

Supplementary Figure 4

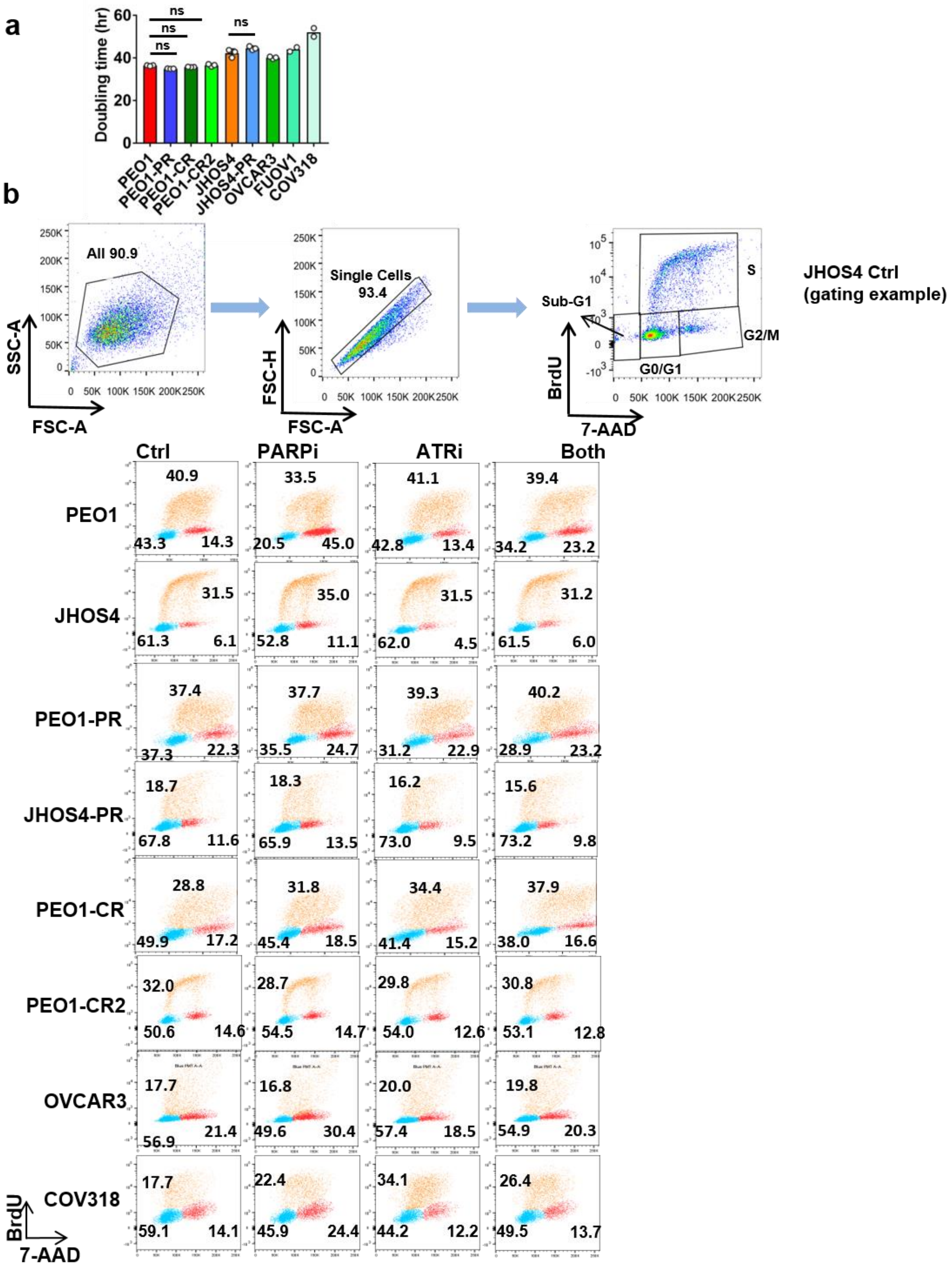

Supplementary Figure 4. Cell cycle evaluation after drug treatment in PARPi and platinum resistant cells. (a) Doubling times were calculated for all cell lines and data shown is Mean ±SD; n=3 independent biological samples, except for FUOV1 and COV318 where n=2. Individual samples are presented as data points. NS=non-significant. **(b)** Gating strategy (for JHOS4 control cells) and representative images of cell cycle analysis by flow cytometry are shown. Parental *BRCA1/2*^{MUT} cells (PEO1, JHOS4) and PARPi and platinum-resistant cells (PEO1-PR, PEO1-CR, JHOS4-PR, PEO1-CR2 and OVCAR3, FUOV1 and CAOV318) were plated and incubated with PARPi 1µM, and ATRi 1µM as well as with combination for 24 hours. Cells were labeled with anti-BrdU and propidium iodide. Population of each phase of cell cycle was calculated; G1 (blue), S (orange), and G2-M (red). Representative data of 3 biological repeats are shown. Source data are provided as a source data file.

Supplemental Figure 5

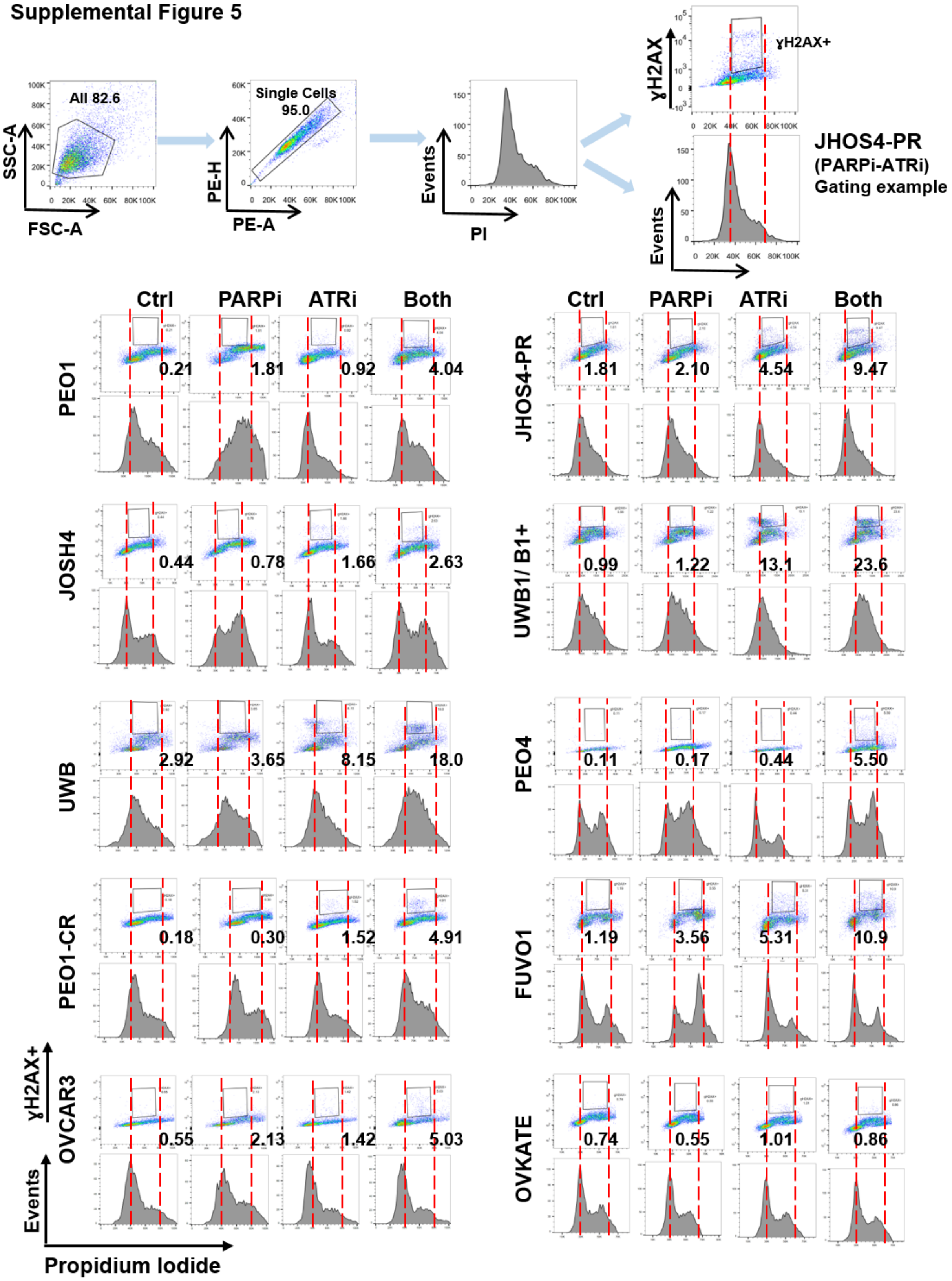

Supplementary Figure 5. Detection of γH2AX in S phase cells after drug treatments. Parental *BRCA1/2*^{MUT} (PEO1, JHOS4, and UWB), PARPi-treatment resistant (PEO1-PR, JHOS4-PR, UWB/B1), and platinum resistant (PEO1-CR, PEO4, OVCAR3 (CCNE1^{High}), FUOV1 (CCNE1^{High}) cells and CCNE1^{low} OVKATE) cells were treated with PARPi (AZD2281), ATRi (AZD6738), as well as with combination of both drugs. JHOS4-PR, OVCAR3 cells were treated with 1μM PARPi, 1μM ATRi (JHOS4-PR for 36 hrs and OVCAR3 for 24 hrs). All other cells were treated at the dosage of 1μM PARPi and 0.5 μM ATRi for 24 hrs. Gating strategy and representative images of analyzing γH2AX positive cells in S phase are shown for JHOS4-PR (PARPi-ATRi treatment). The PI (propidium iodide) histogram showing the G1 (DNA=2n) and G2-M (DNA=4n) phases were lined with scatter images of PI and γH2AX to quantify the γH2AX positive cells at S phase. The representative images of these cell lines (PEO1-PR in Figure 4c) from 3 biological repeats were shown. Source data are provided as a source data file.

Supplementary Figure 6

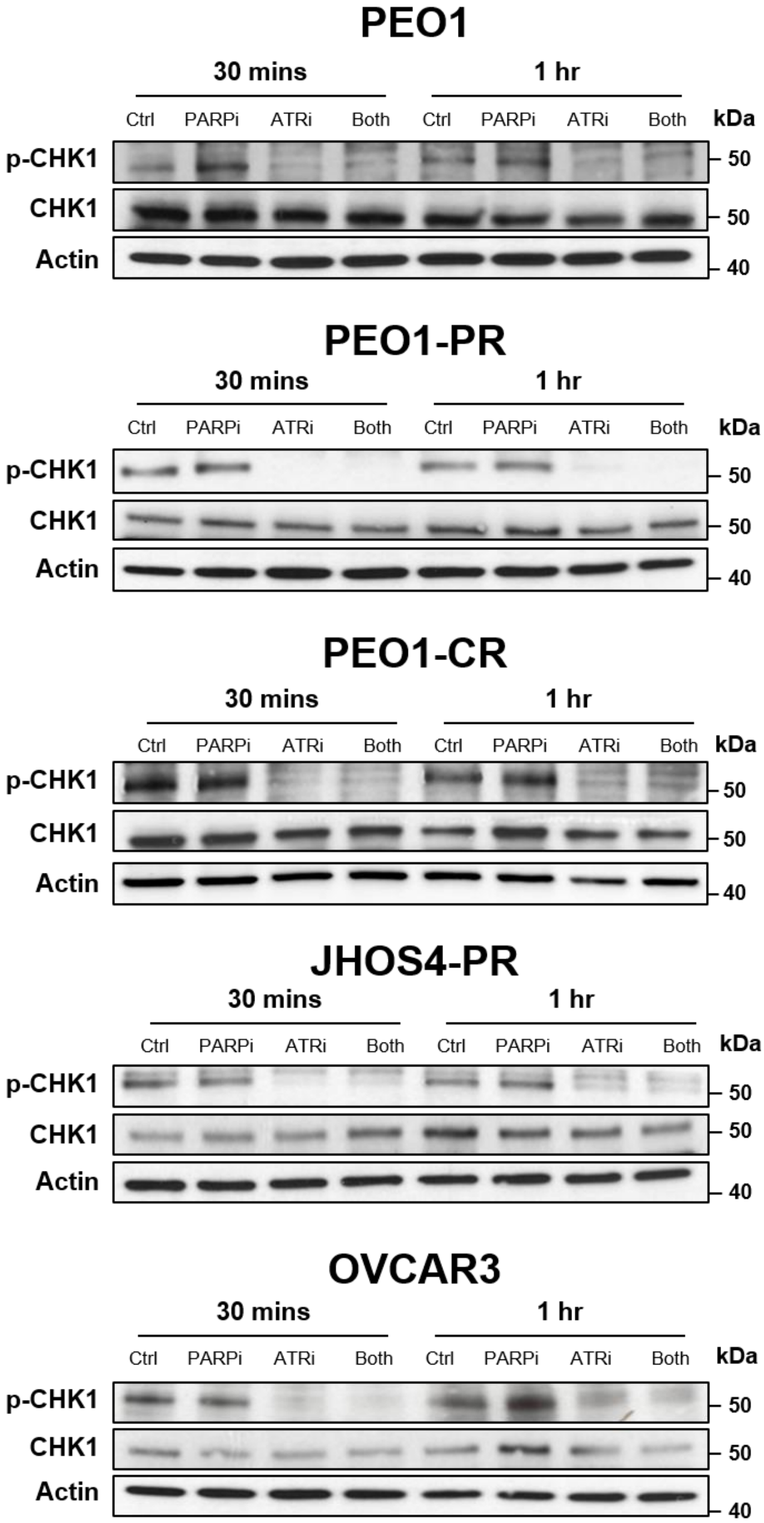

Supplementary Figure 6. Effects of short-term PARPi-ATRi combination treatment on CHK1 signaling. Western blot analysis of parental $BRCA^{MUT}$ (PEO1, $\mathit{BRCA2}^{MUT}$), PARPi-resistant lines (PEO1-PR; JHOS4-PR), and platinum-resistant cell lines (PEO1-CR; $\mathit{CCNE1}^{Amp}$ OVCAR3) cells after the treatment of PARPi (1μM), ATRi (1μM) or both (1μM each) for 30 minutes and one hour. The levels of total CHK1 and p-CHK1 were analyzed by western blotting. Actin was used as the loading control. Representative data of 3 biological assays are shown. Source data are provided as a source data file.

Supplementary Figure 7

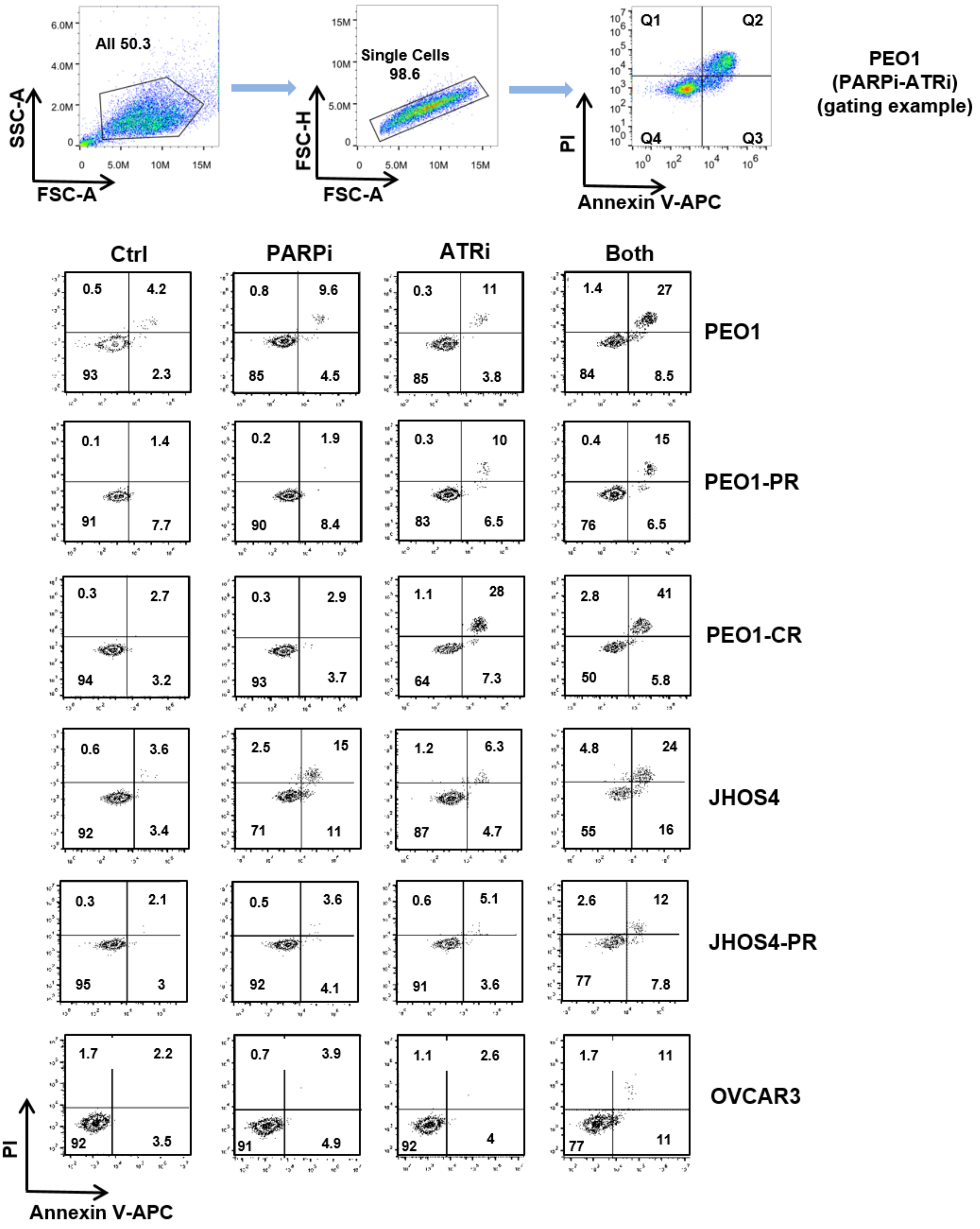

Supplementary Figure 7. Apoptosis analysis after drug treatment in PARPi and platinum-resistant cells. Gating strategy shown that was used to determine the percentage of apoptotic cells in PEO1 cells after PARPi and ATRi treatment. Parental *BRCA1/2*^{MUT} cells (PEO1, JHOS4) and treatment resistant cells (PEO1-PR, PEO1-CR, JHOS4-PR and OVCAR3) were incubated with PARPi (AZD2281) 1µM, ATRi (AZD6738) 1µM, as well as combination of both drugs for 3 days (PEO1, PEO1-PR, PEO1-CR, OVCAR3) and 5 days (JHOS4, JHOS4-PR). Cells were stained with Annexin-V (early apoptosis) and propidium iodide (late apoptosis); apoptotic cell percentage was then measured by flow cytometry. Only Annexin-V positive cells with either both high and low PI signals were counted as an apoptotic cell. Representative data of 3 biological assays are shown. Source data are provided as a source data file.

Supplementary Figure 8

a

WO-2 PDX, *gBRCA2*^{MUT}

Statistical analysis: Tumor Growth

p value	Control	PARPi	ATRi	Both
**PARPi**	0.0264		0.3704	<0.0001
**ATRi**	0.0129	0.3704		<0.0001
**Both**	0.0004	<0.0001	<0.0001	

p value	Control	PARPi	ATRi	Both
**PARPi**	0.0135		0.0382	0.0007
**ATRi**	0.0025	0.0382		0.0007
**Both**	0.0011	0.0007	0.0007	

	Control	PARPi	ATRi	Both
**Median OS (weeks)**	**3**	**10**	**14**	**50.5**

b

WO-57 PDX, *gBRCA1*^{REV}

Statistical analysis: Tumor Growth

p value	Control	PARPi	ATRi	Both
**PARPi**	0.5272		0.4701	0.0452
**ATRi**	0.9418	0.4701		0.0104
**Both**	0.0427	0.0452	0.0104	

p value	Control	PARPi	ATRi	Both
**PARPi**	0.1155		0.2986	0.0292
**ATRi**	0.4366	0.2986		0.0078
**Both**	0.0118	0.0292	0.0078	

	Control	PARPi	ATRi	Both
**Median OS (weeks)**	**6.5**	**10**	**9**	**17**

c

WO-58 PDX, *gBRCA1*^{MUT}

Statistical analysis: Tumor Growth

p value	Control	PARPi	ATRi	Both
**PARPi**	0.5685		0.3367	0.0091
**ATRi**	0.1038	0.3367		0.0320
**Both**	0.0013	0.0091	0.0320	

p value	Control	PARPi	ATRi	Both
**PARPi**	0.3722		0.0372	<0.0001
**ATRi**	0.0371	0.0372		0.0445
**Both**	<0.0001	<0.0001	0.0445	

	Control	PARPi	ATRi	Both
**Median OS (weeks)**	**12**	**9**	**15.5**	**23**

d

WO-19 PDX, *BRCA*^{WT}, *CCNE1*^{AMP}

Statistical analysis: Tumor Growth

p value	Control	Carbo	PARPi	ATRi	Both
**Carbo**	0.4063		0.7178	0.0053	<0.0001
**PARPi**	0.7229	0.7178		0.0084	0.0003
**ATRi**	0.0005	0.0053	0.0084		ns
**Both**	<0.0001	<0.0001	0.0003	ns	

p value	Control	Carbo	PARPi	ATRi	Both
**Carbo**	0.1650		0.1679	0.0626	0.0206
**PARPi**	0.9827	0.1679		0.1025	0.0462
**ATRi**	0.0493	0.0626	0.1025		0.1844
**Both**	0.0142	0.0206	0.0462	0.1844	

	Control	Carboplatin	PARPi	ATRi	Both
**Median OS (weeks)**	**6**	**7**	**7**	**11**	**15**

Supplementary Figure 8. Statistical analysis for tumor growth and survival analysis of PDX pre-clinical trials.

To compare tumor growth curves, longitudinal analysis of tumor growth in PDX experiments was carried out by Linear Mixed Effect modeling on log pre-processed tumor sizes using the TumGrowth web tool (https://kroemerlab.shinyapps.io/TumGrowth/). *P* values were calculated by testing whether tumor growth slopes (on a log scale) were dissimilar between treatment groups with type II ANOVA and pairwise comparisons across groups and presented (Left). Differences in the survival of PDXs was examined by Kaplan-Meier curves with the log-rank test. *P* values are presented (Right). $P<0.05$ was considered statistically significant. **(a)** PARPi resistant *BRCA2*^{MUT} WO-2PR PDX, **(b)** *BRCA1*^{REV} WO-57 PDX, **(c)** *BRCA1*^{MUT} and *CCNE1* CN 7 WO-58 PDX **(d)** platinum resistant *CCNE1*^{Amp} PDX.

Supplementary Figure 9

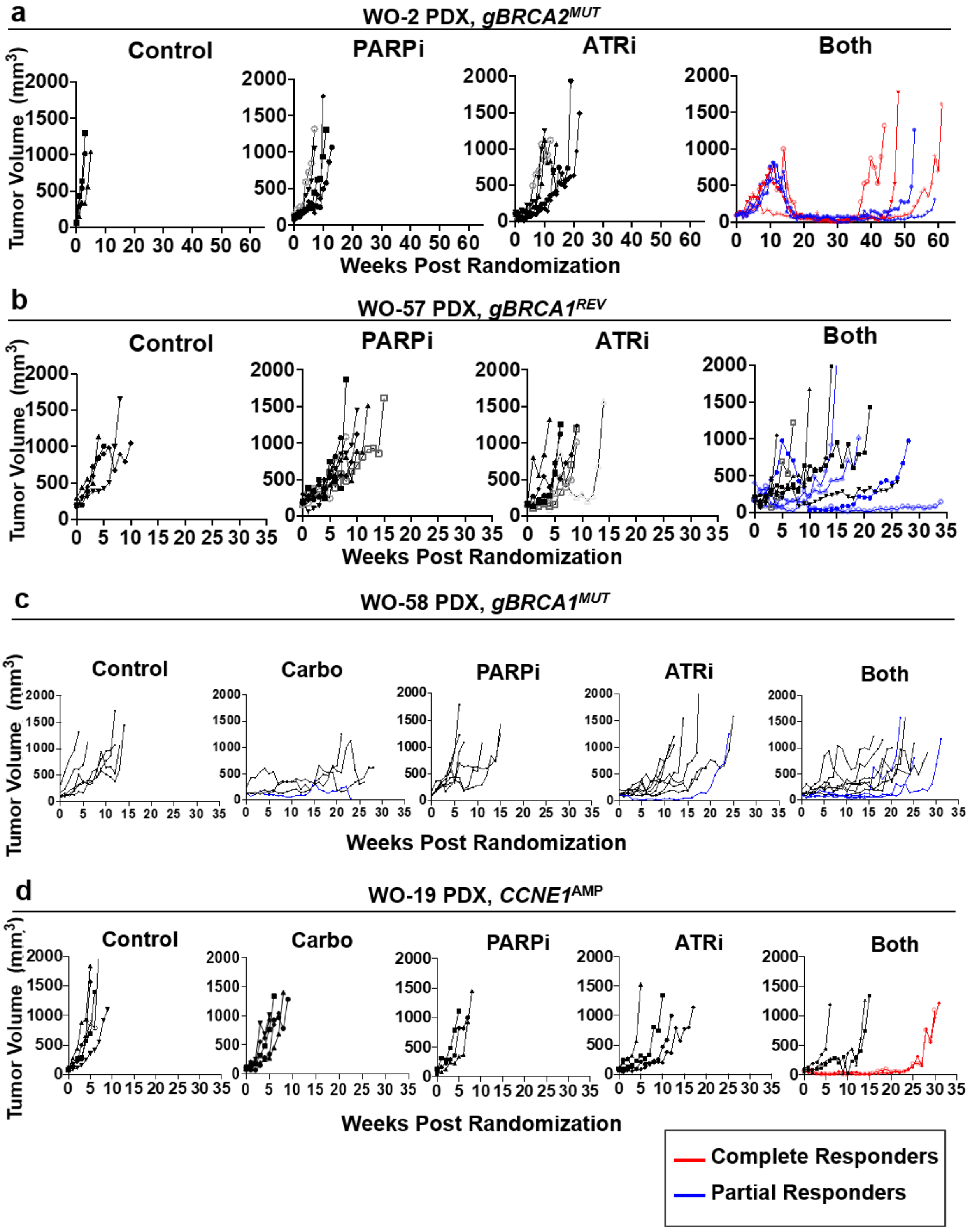

Supplementary Figure 9. Individual PDX tumor growth curves. Tumor growth curve over time of individual mice in each treatment group for each PDX model. Complete (red) and partial responders (blue) are highlighted. **(a)** PARPi-resistant *BRCA2*^{MUT} WO-2PR PDX. Treatment groups: (1) control (*n*=3), (2) PARPi (*n*=5), (3) ATRi (*n*=5), (4) Both (*n*=6) **(b)** PARPi resistant *BRCA1*^{REV} WO-57 PDX. Treatment groups: (1) control (*n*=4), (2) PARPi (*n*=7), (3) ATRi (*n*=7), (4) Both (*n*=10). **(c)** PARPi-resistant *BRCA1*^{MUT}with elevated *CCNE1* copy number (CN=7) and Cyclin E protein overexpression, WO-58 PDX. Treatment groups: (1) control (*n*=6), (2) PARPi (*n*=6), (3) ATRi (*n*=8), (4) Both (*n*=9). **(d)** Platinum and PARPi-resistant *CCNE1*^{Amp} WO-19 PDX. Treatment groups: (1) control (*n*=5), (2) carboplatin (*n*=5), (3) PARPi (*n*=3), (4) ATRi 50 (*n*=4), (5) Both (*n*=6).

Supplementary Figure 10

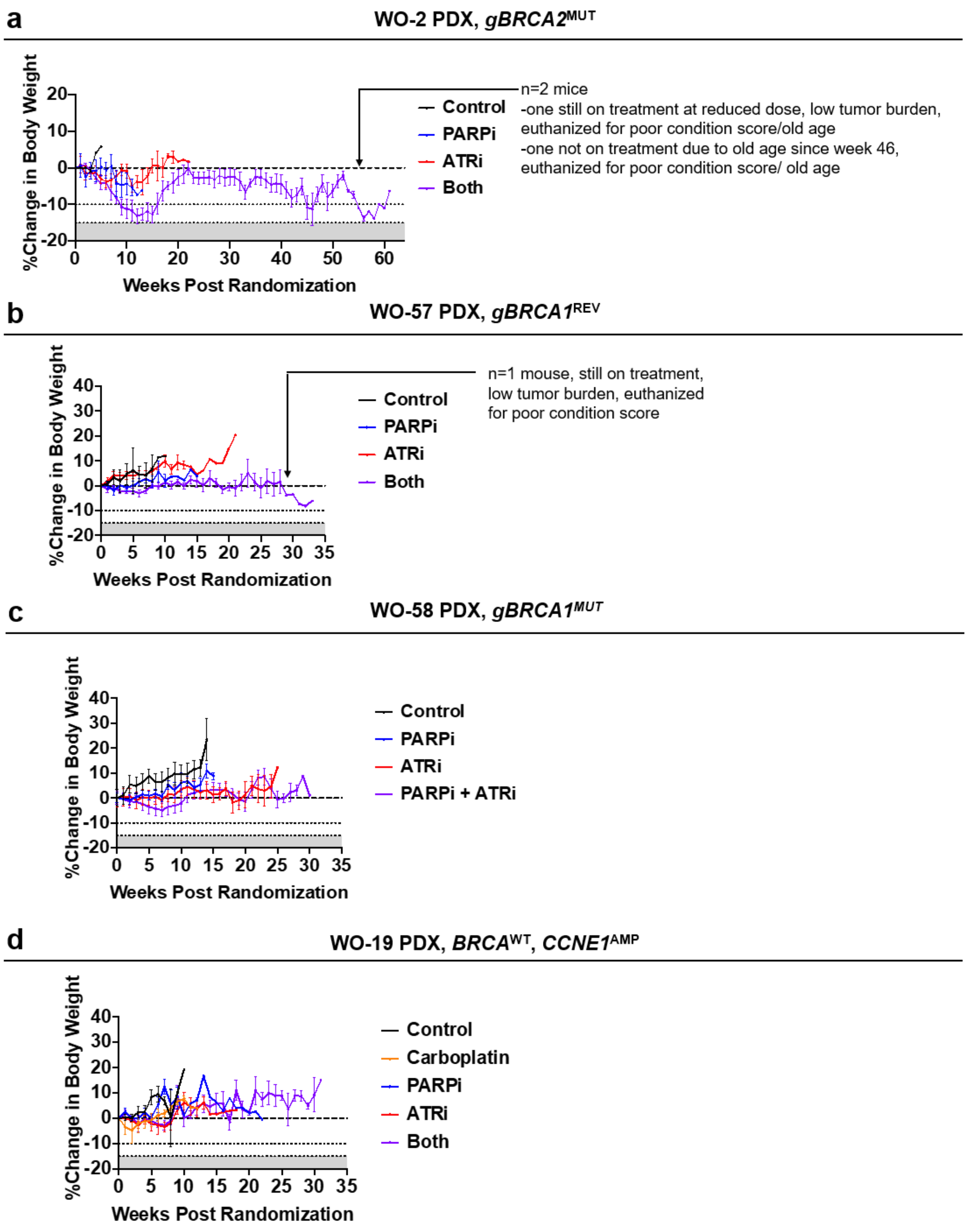

Supplementary Figure 10. Body weights for mice in PDX pre-clinical trials.

Percent change in body weights after randomization into treatment arms. Dashed line at 10% indicates cut-off for resuscitative intervention (-15% was used as cut-off for dose interruption). **(a)** PARPi-resistant *BRCA2*^{MUT} WO-2PR PDX. Treatment groups: (1) control (*n*=3), (2) PARPi (*n*=5), (3) ATRi (*n*=5), (4) Both (*n*=6) **(b)** PARPi resistant *BRCA1*^{REV} WO-57 PDX. Treatment groups: (1) control (*n*=4), (2) PARPi (*n*=7), (3) ATRi (*n*=7), (4) Both (*n*=10). **(c)** PARPi-resistant *BRCA1*^{MUT}with elevated *CCNE1* copy number (CN=7) and Cyclin E protein overexpression, WO-58 PDX. Treatment groups: (1) control (*n*=6), (2) PARPi (*n*=6), (3) ATRi (*n*=8), (4) Both (*n*=9). **(d)** Platinum and PARPi-resistant *CCNE1*^{Amp} WO-19 PDX. Treatment groups: (1) control (*n*=5), (2) carboplatin (*n*=5), (3) PARPi (*n*=3), (4) ATRi 50 (*n*=4), (5) Both (*n*=6). Percent change in body weight from baseline shown is Mean±SEM

Supplementary Figure 11

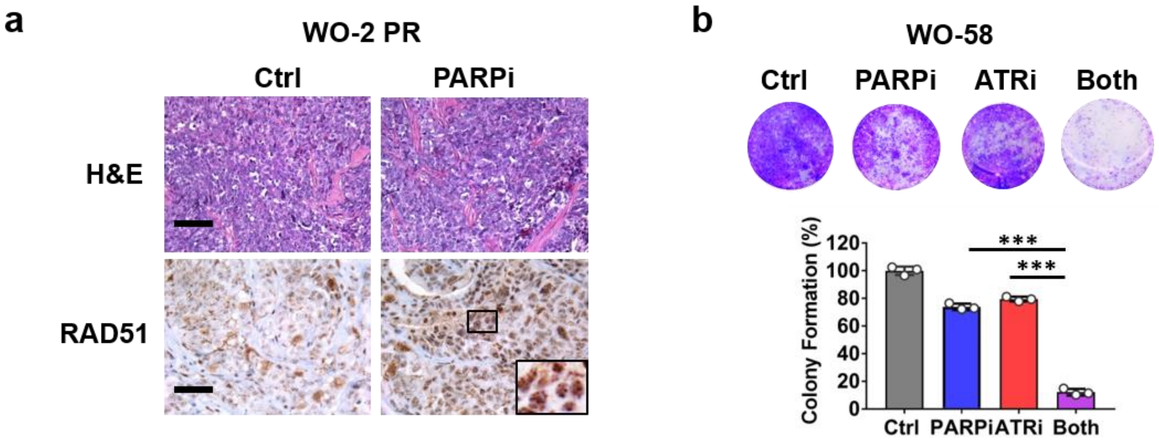

Supplementary Figure 11. Evaluation of PDX models and primary tumor cultures response to PARPi-ATRi

(a) H&E and immunohistochemistry detection of Rad51 foci in PARPi-resistant WO-2 PDX tumors (*BRCA2*^{MUT}). Mice from control and PARPi treatment group (75 mg/kg) were sacrificed 2 weeks post randomization and 6 hours after drug treatment. Representative data of 2 mice in each group is shown. Magnification is X20 for large panels and X40 for inset. Scale bar=50μm. **(b)** Colony formation after WO-58 primary tumor cultures were treated with PARPi (0.5μM), ATRi (0.5μM) and combination for 13 days, and colonies were quantified using ImageJ. One-way ANOVA analysis followed by Tukey's multiple-comparisons test. Data shown as mean±SD (n=3 independent biological samples) of a single representative experiment. ***P<0.0001. Source data are provided as a source data file.

Supplementary Table 1.

Cell Line	*BRCA* germline status	Mutations in biologically relevant genes in all cell lines	New mutations in any resistant cell lines	Loss of heterozygosity (LOH)	CNA
PEO1	***BRCA2*** **c.6620C>G, p.Tyr1655***	***CREBBP, TP53***	**-**	***BRCA2*** **LOH**	**CDKN2A, NF2, WWOX homozygous deletions**
PEO1-PR	***BRCA2*** **c.6620C>G, p.Tyr1655***	***CREBBP, TP53***	**9 mutations;** ***COL3A1, ^^STAG2, ^HERC2, KRTAP4-3, OR9G1, UQCRFS1, CCDC15, PABPC1, ^PRKDC***	***BRCA2*** **LOH**	**CDKN2A, NF2, WWOX homozygous deletions**
PEO1-PR1	***BRCA2*** **c.6620C>G, p.Tyr1655***	***CREBBP, TP53***	**6 mutations;** ***COL3A1, ^^STAG2, ROS1, ARMC4, ^EXO1, GXYLT1,***	***BRCA2*** **LOH**	**CDKN2A, NF2, WWOX homozygous deletions**
PEO1-PR2	***BRCA2*** **c.6620C>G, p.Tyr1655***	***CREBBP, TP53***	**5 mutations;** ***^^STAG2, ^^^ANKRD30A, ^^^ANKRD30B, CNTN5***	***BRCA2*** **LOH**	**CDKN2A, NF2, WWOX homozygous deletions**
PEO1-CR	***BRCA2*** **c.6620C>G, p.Tyr1655***	***CREBBP, TP53***	**5 mutations;** ***COL3A1, CNTN5, KRTAP4-3, PLCE1, TNFAIP3,***	***BRCA2*** **LOH**	**CDKN2A, NF2, WWOX homozygous deletions**
PEO1-CR1	***BRCA2*** **c.6620C>G, p.Tyr1655***	***CREBBP, TP53***	**9 mutations;** ***COL3A1, CNTN5, KRTAP4-3, UQCRFS1, ^HERC2, OR9G1, GXYLT1 GNAS, SDHC,***	***BRCA2*** **LOH**	**CDKN2A, NF2, WWOX homozygous deletions**
PEO1-CR2	***BRCA2*** **c.6620C>G, p.Tyr1655***	***CREBBP, TP53***	**5 mutations;** ***COL3A1,^^^RARA, RELN, RNF43, GXYLT1***	***BRCA2*** **LOH**	**CDKN2A, NF2, WWOX homozygous deletions**
JHOS4	***BRCA1*** **c.5341-1G>A Splice_acceptor**	***TP53***	**-**	***BRCA1*** **LOH**	
JHOS4-PR	***BRCA1*** **c.5341-1G>A Splice_acceptor**	***TP53***	**5 mutations;** ***HLA-B, PABPC1, TAS2R43, RECQL5, ^XPC***	***BRCA1*** **LOH**	***XPC amplification***
JHOS4-PR1	***BRCA1*** **c.5341-1G>A Splice_acceptor**	***TP53***	**3 mutations;** ***TAS2R43, ACVR1B, ^XPC***	***BRCA1*** **LOH**	***XPC amplification***
JHOS4-PR2	***BRCA1*** **c.5341-1G>A Splice_acceptor**	***TP53***	**3 mutations;** ***^^^HOMEZ, MUC16, ^XPC***	***BRCA1*** **LOH**	***XPC amplification***

Supplementary Table 1. Gene mutation profile in drug resistant cell lines

Parental *BRCA1/2*^{MUT} cells (PEO1, JHOS4), acquired PARPi-resistant cells (PEO1-PR, PEO1-PR1, PEO1-PR2, JHOS4-PR, JHOS4-PR1, JHOS4-PR2), and carboplatin-resistant cell lines (PEO1-CR, PEO1-CR1, PEO1-CR2) were analyzed by whole genome sequencing. Pathogenic mutations in biologically relevant genes including *BRCA1/2* in each cell line were shown. New pathogenic mutations, and copy number alterations found in any resistant cell lines including clonal cell lines were listed. (^Genes involved in DNA repair, ^^DNA replication, and ^^^Transcription, ^*XPC* amplified).

Supplementary Table 2.

Sample	BRCA germline status	Mutations in biologically relevant genes	New mutations in resistant PDX	Loss of Heterozygosity (LOH)	*CNA*
**WO-2 Patient tumor**	***BRCA2* E2906Gfs*12 (germline)**	***TP53***	**-**	***BRCA2* LOH**	***PPP2R1 hemizygous deletion***
**WO-2 PDX tumor (PARPi resistant)**	***BRCA2* E2906Gfs*12 (germline)**	***TP53***	**22 mutations; *CAMKMT, DNAAF1, PLCB1, SF3A3, SLC2A6, SLC4A11, TTC23L, VTI1A, AMPD1, CILP, COL2A1, DENND5B, DNAH17, DTHD1, ^EXO1, IL2RG, NUDCD1, PXMP4, SRPRB, TALDO1, JAG1, HNRNPDL***	***BRCA2* LOH**	
**WO-57 Patient tumor (PARPi resistant)**	***BRCA1* I815Ffs*31**	***BRCA1* H839Qfs*12 (reversion mutation; puts gene back in frame in combination with germline frameshift) , *TP53***	**-**	***BRCA1* LOH**	***RB1 homozygous deletion***
**WO-57 PDX tumor (PARPi resistant)**	***BRCA1* I815Ffs*31**	***BRCA1* H839Qfs*12 (reversion mutation;puts gene back in frame in combination with germline frameshift) , *TP53***	**-**	***BRCA1* LOH**	
**WO-58 Patient tumor (PARPi Resistant)**	***BRCA1* L625V**	***TP53***	**-**	***BRCA1* LOH**	***CCNE1* Copy number 7**
**WO-58 PDX (PARPi Resistant)**	***BRCA1* L625V**	***TP53***	***CLDN17, EFCAB4B, F10, SSRP1***	***BRCA1* LOH**	***CCNE1* Copy number 7**
**WO-19 Patient tumor (Carboplatin resistant)**	***BRCA1 /2* Wild Type**	***TP53***	**-**	**-**	***CCNE1* Copy number 16**
**WO-19 PDX tumor (Carboplatin resistant)**	***BRCA1 /2* Wild Type**	***TP53***	**-**	**-**	***CCNE1* Copy number 20**

Supplementary Table 2: Gene mutation profile in drug resistant PDX models.

Mutations in three PARPi resistant models (WO-57 *BRCA1*^{REV}, WO-58 *BRCA1*^{MUT} CCNE1 CN 7, and WO-2 *BRCA2*^{MUT}) and a platinum resistant model (WO-19 *CCNE1* amplified) were analyzed by whole exome sequencing. Mutations in biologically relevant genes including *BRCA1/2* in each PDX models were shown. New mutations found in resistant PDXs were listed. WO-2 PARPi resistant PDX retained original germline mutations as well as LOH in *BRCA2* genes. *BRCA1* reversion mutation found in WO-57 patient tumor and PDX tumor. WO-58 retained original BRCA1 mutation and had a *CCNE1* copy number of 7. **^**genes involved in DNA repair.

Supplementary Table 3. List of antibodies

FLOW CYTOMETRY

Antibody	Fluorochrome	Dilution	Company
Gamma-H2AX	APC	1:300	Cell Signaling Technology(CST), #9718S

WESTERN BLOT, IHC, IF, BLOCKING

Antibody	IHC/IF	WB	Lot#	Details
p-ATR	-	1:750	9	CST, #2853,
ATR	-	1:1000	4	CST, #13934
p-CHK1		1:750	18	CST, #2348
CHK1	-	1:1000	3	CST, 2360
Beta-ACTIN	-	1:10000	17	CST, #3700
GAPDH	-	1:10000	14	CST, #2118
53BP1	-	1:1000	090419	Novus Biological, #NB100-904
53BP1	-	1:1000	5	CST, #4937, Polyclonal
MDR1	-	1:1000	K2117	Santa Cruz Biotechnology, #sc 55510, Clone-D11
Artemis	-	1:1000	1	CST, 13381, Clone-D7O8V
XPC	-	1:1000	1	CST, #14768
BRCA2 (N-ter)	-	1:5000	M 104074	Bethyl Laboratory, #A303-434A , Polyclonal
BRCA2 (C-ter)	-	1:5000	M 104074	Bethyl Laboratory, #A303-435A , Polyclonal
HSP90	-	1:1000	1	CST, #4874, Polyclonal
Ku80	-	1:1000	1	CST, #2180, Clone- C48E7
RIF1	-	1:1000	4	Bethyl Laboratory, #A300-568A , Polyclonal
PTIP	-	1:1000	2	Abcam, ab70434, Polyclonal
RAD51	1:400	-	J0215	Santa Cruz Biotechnology, #sc 8349, Clone-H92
Caspase-3	1:300	-	21	CST, #9664, Clone-5A1E
Gamma-H2AX	1:500	-	17	CST, #9718, Clone-20E3
Geminin	1:1000		1	CST, #52508

Supplementary Methods

In vitro cytotoxicity assays

Cells (5 x 10^3) were seeded on 96-well plates and treated with the indicated doses of PARPi (AZD2281, AstraZeneca, Wilmington, DE), ATRi (AZD6738, AstraZeneca, Wilmington, DE) and carboplatin (Hospira Inc., King of Prussia, PA) for 5 days. At the end of the treatment period, the relative cell viability was determined by an MTT colorimetric assay (Sigma Aldrich Co., St Louis, MO). IC_{50} curves were generated using GraphPad Prism (GraphPad Software, San Diego, CA).

Colony formation assays

Cells (1-2 x 10^4) were plated onto 12-well plates and incubated at 37°C. Cells were treated with the indicated doses of PARPi, ATRi and carboplatin for 10-14 days. Media and drugs were refreshed every 3-4 days. Colonies were stained with 0.2% crystal violet. Whole well images were scanned and colony forming area was quantitated using ImageJ (NIH, Bethesda, MD).

Cell-cycle analysis

Cell cycle was analyzed using a FITC-BrdU Flow Kit (BD Biosciences, San Jose, CA). Cells were incubated with drugs (24 hrs) and Bromodeoxyuridine (BrdU; 10 µM) was added for 2 hours before harvest. Cells were labeled with FITC-conjugated anti-BrdU and propidium iodide (PI) solution and analyzed by flow cytometry (BD FACSCalibur, BD Biosciences, San Jose, CA). Data was analyzed by FlowJo software (Tree Star, Inc., Ashland, OR).

Apoptosis analysis

Cells were incubated with drugs for 3-5 days. Apoptosis was detected by using an Annexin V Flow Kit (BD Biosciences, Franklin Lakes, NJ) according to the manufacturer's instructions. Annexin V-labeled cells were analyzed by flow cytometry (FACS Calibur; BD Biosciences, San Jose, CA). The data was analyzed by FlowJo software (Tree Star, Inc., Ashland, OR).

Apoptosis was evaluated by western blot with cleaved-Caspase3 antibody (Supplementary Table 3).

Immunofluorescence

Cells were seeded on a coverslip and then incubated with drugs for 24 hours and fixed with 4% paraformaldehyde (PFA). Cells were stained with RAD51 and Geminin antibody (Supplementary Table 3). Fluorescence images were obtained with microscope with filter wheels for excitation and emission of DAPI, FITC, and Cy3 fluorescent dyes (LI-COR Biotechnology). Images were quantitatively assessed using ImageJ software (NIH, Bethesda, MD), along with the ND2 plugin.

Immunohistochemistry

Tissue samples were fixed in 10% formalin and embedded in paraffin. Paraffin blocks were cut into 4- to 6-μm sections and placed onto slides. After deparaffinization and rehydration, antigen retrieval was performed. Slides were incubated with pCHK1, cleaved-caspase 3, and γH2AX antibody (Supplementary Table 3). Slides were incubated with anti-rabbit HRP and developed using 3,3′-diaminobenzidine (DAB) and chromogen.

Assessment of IHC staining was performed following preparation of high-quality digital whole-slide images using a Leica Biosystems Aperio scanning microscope (Leica Biosystems Inc., Buffalo Grove, IL). Whole slide images were then fragmented into multiple high power images and quantitatively assessed using ImageJ software (NIH, Bethesda, MD), along with the ImmunoRatio plugin. Percent nuclear staining was measured for markers cleaved-caspase 3, pCHK1, and γH2AX (Supplementary Table 3). The resulting percent values were averaged to give a mean value for each treatment group.
