# Supplementary material for: Combining PARP with ATR inhibition overcomes PARP inhibitor and platinum resistance in ovarian cancer models
Source: Nat Commun. 2020 Jul 24;11:3726. doi: 10.1038/s41467-020-17127-2 (PMC7381609; doi:10.1038/s41467-020-17127-2)
Supplement: Supplementary file 2 — Reporting Summary [file 41467_2020_17127_MOESM2_ESM.pdf]

nature research

Corresponding author(s): Fiona Simpkins, MD

Last updated by author(s): 5/28/2020

Reporting Summary

Nature Research wishes to improve the reproducibility of the work that we publish. This form provides structure for consistency and transparency in reporting. For further information on Nature Research policies, see Authors & Referees and the Editorial Policy Checklist .

Statistics

For all statistical analyses, confirm that the following items are present in the figure legend, table legend, main text, or Methods section.

n/a	Confirmed	
☐	☒	The exact sample size (*n*) for each experimental group/condition, given as a discrete number and unit of measurement
☐	☒	A statement on whether measurements were taken from distinct samples or whether the same sample was measured repeatedly
☐	☒	The statistical test(s) used AND whether they are one- or two-sided<br>*Only common tests should be described solely by name; describe more complex techniques in the Methods section.*
☐	☒	A description of all covariates tested
☐	☒	A description of any assumptions or corrections, such as tests of normality and adjustment for multiple comparisons
☐	☒	A full description of the statistical parameters including central tendency (e.g. means) or other basic estimates (e.g. regression coefficient) AND variation (e.g. standard deviation) or associated estimates of uncertainty (e.g. confidence intervals)
☐	☒	For null hypothesis testing, the test statistic (e.g. *F*, *t*, *r*) with confidence intervals, effect sizes, degrees of freedom and *P* value noted<br>*Give P values as exact values whenever suitable.*
☒	☐	For Bayesian analysis, information on the choice of priors and Markov chain Monte Carlo settings
☒	☐	For hierarchical and complex designs, identification of the appropriate level for tests and full reporting of outcomes
☒	☐	Estimates of effect sizes (e.g. Cohen's *d*, Pearson's *r*), indicating how they were calculated

Our web collection on statistics for biologists contains articles on many of the points above.

Software and code

Policy information about availability of computer code

Data collection	Microsoft excel (vs 2016), Ultrasound measurements by Sonosite imager (Edge II Ultrasound System), MTT calorimetric assay software (Gen5 ELISA, v 1.04.5), FiberVision scanner.
Data analysis	GraphPad Prism (virsion 8.4.2), ImageJ (1.50i), Flow Jo (version 10), Genomic Vision FiberStudio v2.0, Strelka v2.9.0, Manta v1.3.1, TumGrowth web tool (no version available), VariantDx (no version available; PMID: 25877891), ELAND (version 1.8.2), Tumor Manager (v 3.3.4)

For manuscripts utilizing custom algorithms or software that are central to the research but not yet described in published literature, software must be made available to editors/reviewers. We strongly encourage code deposition in a community repository (e.g. GitHub). See the Nature Research guidelines for submitting code & software for further information.

Data

Policy information about availability of data

All manuscripts must include a data availability statement. This statement should provide the following information, where applicable:

- Accession codes, unique identifiers, or web links for publicly available datasets
- A list of figures that have associated raw data
- A description of any restrictions on data availability

All whole genome and exome sequencing data generated for this study have been deposited in the NCBI GEO database (https://www.ncbi.nlm.nih.gov/gds/) under accession # PRJNA626435 and PRJNA626436. The source data underlying Figures 1a-b, 1d, 2a-g, 3a-c, 4a-c, 5c, 5e-h, 6a-g, Supplementary Figures 1a-f, 2a-d, 3a-b, 4a-b, 5, 6, 7, 8a-d, 9a-d, 10 a-d, 11b, and Supplementary Tables 1, 2 and 3 are provided as a Source Data File. All the other data supporting the findings of this study are available within the article and its supplementary information files and from the corresponding author upon reasonable request.

Field-specific reporting

Please select the one below that is the best fit for your research. If you are not sure, read the appropriate sections before making your selection.

[x] Life sciences [ ] Behavioural & social sciences [ ] Ecological, evolutionary & environmental sciences

For a reference copy of the document with all sections, see nature.com/documents/nr-reporting-summary-flat.pdf

Life sciences study design

All studies must disclose on these points even when the disclosure is negative.

Sample size

The statistical power for animal study is characterize in terms of effect size, defined as A/B, where A = the difference in mean tumor volume at a specific time in control mice compared to each of the treated groups, and B = the estimated pooled variance (in controls and treated mice) for the two groups being compared. An effect size over 1.0 is generally considered to be large, but can be expected to occur in well-controlled studies of treatments with relatively large effects on biological systems. With N = 10 mice per group, the probability is 0.56 of finding a statistically significant difference when treatment induces a difference of 1.0 standard deviation, and the probability is 0.89 of finding a statistically significant difference when treatment induces a difference of 1.5 standard deviations. To ensure statistical power in our results, we transplanted 12 mice per arm. After randomization, the majority of arms had 10 mice per arm with range 4-10.

For In vitro studies, "n" was chosen based on prior publications. No statistical model was used to predetermine the sample size. For in vitro experiments, 3-5 biological samples were tested in at least 3 independent experiments to draw a conclusion except for DNA combing studies where 2 independent experiments were performed. Two slides per condition were evaluated.

Data exclusions

For in vitro studies, no data was excluded. For in vivo studies, mice that died for an unknown reason (low tumor burden, stable and normal weights, normal condition scores) would be excluded from analysis.

Replication

All in vitro studies were performed at least in three independent experiments. All conditions in each experiment had 3-5 biological replicates. All attempts at replication for in vitro experiments were successful. PDX expts were not repeated using the same model as this would not be acceptable by IUCUC.

Randomization

For animal studies, mice were randomized to treatment groups using Tumor Manager (v 3.3.4) randomizing software by Biopticon. For In Vitro study, 3 biological replicates were randomly assigned to treatment groups. Cell cultures were randomly assigned to different groups or treatments.

Blinding

For animal studies, weekly ultrasound measurements, weight assessments, and condition scores were obtained for treatment groups in a blinded manner. Histology was reviewed by pathologists and scored in a blinded manner.

Reporting for specific materials, systems and methods

We require information from authors about some types of materials, experimental systems and methods used in many studies. Here, indicate whether each material, system or method listed is relevant to your study. If you are not sure if a list item applies to your research, read the appropriate section before selecting a response.

Materials & experimental systems

n/a	Involved in the study	
[ ]	[x]	Antibodies
[ ]	[x]	Eukaryotic cell lines
[x]	[ ]	Palaeontology
[ ]	[x]	Animals and other organisms
[ ]	[x]	Human research participants
[x]	[ ]	Clinical data

Methods

n/a	Involved in the study	
[x]	[ ]	ChIP-seq
[ ]	[x]	Flow cytometry
[x]	[ ]	MRI-based neuroimaging

Antibodies

Antibodies used

phospho-ATR (cat. # ABE462, Lot# Q2475126, EMD Millipore, Billerica, MA),
total ATR (cat. # sc1887, Lot# A1515, Santa Cruz Biotechnology, Inc., Dallas, TX)
phospho-CHK1 (Ser345; cat. # 2348, Lot# 18, Cell Signaling Technology, Inc., Danvers, MA)
total CHK1 (cat. # sc8408, Lot# I2515, Santa Cruz Biotechnology, Inc., Dallas, TX)
γH2AX (cat. # 9718, Lot # 17, Cell Signaling Technology, Inc., Danvers, MA)
MDR1 (cat. # 13342, Lot# 1, Cell Signaling Technology, Inc., Danvers, MA)
Anti-β-Actin (cat. # 3700, Lot# 15, Cell Signaling Technology, Inc., Danvers, MA)
Artemis (cat. # 13381, Lot# 1, Cell Signaling Technology, Inc., Danvers, MA)
Rad51 (cat. # PC130, Lot# 3045546, Millipore Sigma, Burlington, MA)

Ku80 (cat. # 2180, Lot# 1, Cell Signaling Technology, Inc., Danvers, MA)
53BP1 (cat. # 4937, Lot# 5, Cell Signaling Technology, Inc., Danvers, MA)
Rif1 (cat. # A300-568A-M, Lot# 4, Bethyl Laboratories, Montgomery, Texas)
PTIP (cat. # A300-370A-M, Lot# 2, Bethyl Laboratories, Montgomery, TX)
BRCA2 N-term (cat. # A303-434A, Bethyl Laboratories, Montgomery, TX)
BRCA2 C-term (cat. # A303-435A, Bethyl Laboratories, Montgomery, TX)
XPC (cat. # 14768, Lot# 1, Cell Signaling Technology, Inc., Danvers, MA)
MDR1 (cat. # sc55510, Santa Cruz Biotechnology, Inc., Dallas, TX)
HSP-90 (cat. # 4874, Polyclonal, Lot# 1, Cell Signaling Technology, Inc., Danvers, MA)
Caspase3 (cat. # 9664, Clone 5A1E, Lot# 21, Cell Signaling Technology, Inc., Danvers, MA)
Geminin (cat. # 52508, Lot 1, Cell Signaling Technology, Inc., Danvers, MA)

All Antibody information also found in the Supplementary Table 3 "List of Antibody".

Validation

p-ATR (Cell Signaling Tech. (CST), #2853) validated in western blot analysis of Raw264.7, SV-T2 and HT-29 cells that were untreated or UV-treated (50 mJ, 30 min), using Phospho-ATR (Ser428) Antibody by CST. They used Lambda phosphatase NEB #P0753 (10,000 Units/ml, 1hr) to demonstrate the phospho-specificity of the antibody.
ATR (CST, #13934) validated in western blot analysis of extract from various cell lines by CST.
p-CHK1 (CST, #2348) validated in western blot analysis of extract from HeLa, COS, NIH/3T3 and C6 cells, untreated or UV-treated, using Phospho-Chk1 (Ser345) (133D30) Rabbit mAb by CST.
CHK1 (CST, 2360) validated in western blot analysis of extract from various cell lines by CST.
Beta-ACTIN (CST, #3700) validated in western blot analysis of extract from various cell lines by CST.
GAPDH (CST, #2118) validated in western blot analysis of extract from various cell lines by CST.
53BP1 (Novus Biologicals, #NB100-904) validated in western blot analysis of extract from U2OS and 2780 cell lines by Novus Biologicals.
53BP1 (CST, #4937, Polyclonal) validated in western blot analysis of extract from HT29 cell line by CST.
MDR1 (Santa Cruz Biotechnology, #sc55510) validated in western blot analysis of extract from various cell lines by Santa Cruz Biotechnology Inc.
Artemis (CST, 13381) validated in western blot analysis of extract from various cell lines by CST.
XPC (CST, #14768) validated in western blot analysis of extract from various cell lines by CST.
BRCA2 (N-ter) (Bethyl Laboratory, #A303-434A) validated in western blot analysis of extract from various cell lines by Bethyl Laboratory.
BRCA2 (C-ter) (Bethyl Laboratory, #A303-435A) validated in western blot analysis of extract from HeLa cell line by Bethyl Laboratory.
HSP90 (CST, #4874) validated in western blot analysis of extract from various cell lines by CST.
Ku80 (CST, #2180) validated in western blot analysis of extract from various cell lines by CST.
RIF1 (Bethyl Laboratory, #A300-568A) validated in western blot analysis of extract from HeLa and HEK293T cell lines by Bethyl Laboratory.
PTIP (Abcam, ab70434) validated in western blot analysis of extract from HEK293T cell line by Abcam.
RAD51 (Santa Cruz Biotechnology, #sc8349) validated in IF analysis published by Onyango et al. 2016 (PMID- 27084940).
Caspase-3 (CST, #9664) validated in IHC analysis using paraffin-embedded mouse embryo by CST.
Gamma-H2AX (CST, #9718) validated in western blot analysis of extract from untreated and UV-treated 293 cells by CST.
Geminin (CST, #52508)-validated in IHC analysis using paraffin-embedded human endometrioid adenocarcinomaby CST.

Eukaryotic cell lines

Policy information about cell lines

Cell line source(s)

PEO1 and PEO4 was a gift from Dr. Andrew Godwin, University of Kansas and are not commercially available. PEO1-PR, PEO1-CR, JHOS4-PR were generated after 1 year continuous culture in olaparib. UWB 1.289/53PB1 KO cell line was provided by Dr. Roger Greenberg, University of Pennsylvania, Philadelphia and is not comercially available. WO-58 primary cell line was derived from our WO-58 PDX model. The remaining cell lines (e.g. OVCAR3) were obtained from ATCC.
Kuramochi, OVKATE cell lines are commercially available from Japanese Collection of Research Bioresources Cell Bank.

Authentication

All cell lines authentication was evaluated by Short Tandem Repeat (STR) analysis condcted by Wistar Genomics Core

Mycoplasma contamination

All cell lines tested negative for mycoplasma contamination by PCR analysis by Wistar Genomics Core

Commonly misidentified lines
(See ICLAC register)

No cell lines used in this paper are listed in the database of commonly misidentified cell lines (NCBI Biosample)

Animals and other organisms

Policy information about studies involving animals; ARRIVE guidelines recommended for reporting animal research

Laboratory animals

NSG mice (NOD/SCID IL2Rγ−/−), Female, 8 week old.

Wild animals	Study did not involve wild animals
Field-collected samples	The study did not involve samples collected from the field
Ethics oversight	Institutional Animal Care and Use Committee and IRB at UPENN provided guidance on the study protocol.

Note that full information on the approval of the study protocol must also be provided in the manuscript.

Human research participants

Policy information about studies involving human research participants

Population characteristics	*Describe the covariate-relevant population characteristics of the human research participants (e.g. age, gender, genotypic information, past and current diagnosis and treatment categories). If you filled out the behavioural & social sciences study design questions and have nothing to add here, write "See above."*
Recruitment	*Describe how participants were recruited. Outline any potential self-selection bias or other biases that may be present and how these are likely to impact results.*
Ethics oversight	*Identify the organization(s) that approved the study protocol.*

Note that full information on the approval of the study protocol must also be provided in the manuscript.

Flow Cytometry

Plots

Confirm that:

- [x] The axis labels state the marker and fluorochrome used (e.g. CD4-FITC).
- [x] The axis scales are clearly visible. Include numbers along axes only for bottom left plot of group (a 'group' is an analysis of identical markers).
- [x] All plots are contour plots with outliers or pseudocolor plots.
- [x] A numerical value for number of cells or percentage (with statistics) is provided.

Methodology

Sample preparation	Cell cycle was analyzed using a FITC-BrdU Flow Kit (BD Biosciences, San Jose, CA). Cells were dissociated with trypsin and labeled with FITC-conjugated anti-BrdU and propidium iodide (PI) solution.<br>Apoptosis was detected by using an Annexin V Flow Kit (BD Biosciences, Franklin Lakes, NJ). Cells were dissociated with trypsin and labeled with APC-conjugated anti-Annexin V.
Instrument	BD LSR II, a four laser flow cytometry machine (BD Bioscience)
Software	We used FlowJo version 10 to analyze data.
Cell population abundance	Flow cytometry was used for quantification analysis only, no post sorting fractions were collected
Gating strategy	The cell debris were opt-out based on the FSC/SSC. Only the viable cells were analyzed. Singlet and doublet cells were discriminated using FSC-A/ FSC-W gating. The negative control group was used as a comparison to gate the percentage of positive cells.

- [x] Tick this box to confirm that a figure exemplifying the gating strategy is provided in the Supplementary Information.
